# Design and Validation of a New Tilting Rotor VTOL Drone: Structural Optimization, Flight Dynamics, and PID Control

**DOI:** 10.3390/s25113537

**Published:** 2025-06-04

**Authors:** Haixia Gong, Wei He, Shuping Hou, Ming Chen, Ziang Yang, Qin Si, Deming Zhao

**Affiliations:** Mechanical and Electrical Engineering College, Harbin Engineering University, Harbin 150001, China; houshuping@hrbeu.edu.cn (S.H.); chenm@hrbeu.edu.cn (M.C.); yza020214@hrbeu.edu.cn (Z.Y.); 98322252@hrbeu.edu.cn (Q.S.); s321077084@hrbeu.edu.cn (D.Z.)

**Keywords:** tilt-rotor VTOL UAV, structural optimization, vibration analysis, dual-serial PID control, flight dynamics, experimental validation

## Abstract

This study addresses the gap in the experimental validation of the tilt-rotor vertical take-off and landing (VTOL) UAVs by developing a novel prototype that integrates fixed-wing and multi-rotor advantages. A dynamic model based on the “X” quadrotor configuration was established, and Euler parameters were employed to derive the attitude transformation matrix. Structural optimization using hybrid meshing and inertia release methods revealed a maximum deformation of 57.1 mm (2.82% of half-wingspan) and stress concentrations below material limits (379.21 MPa on fasteners). The landing gear was optimized using the unified objective method, and the stress was reduced by 32.63 MPa compared to the pre-optimization stress. Vibration analysis identified hazardous frequencies (11–12 Hz) to avoid resonance. Stable motor speed tracking (±5 RPM) and rolling attitude control (less than 10% error) are achieved using a dual-serial PID control system based on the DSP28377D master. Experimental validation in low-altitude flights confirmed the prototype’s feasibility, though ground effects impacted pitch/yaw performance. This work provides critical experimental data for future tilt-rotor UAV development.

## 1. Introduction

The maximum speed of conventional helicopters is limited by aerodynamic constraints, installed engine power, and airframe drag [1], and problems related to installed engine power and airframe drag can be minimized by careful design, but the main factor limiting the maximum speed of helicopters is the backward paddle stall, and conventional fixed-wing UAVs require a long flight distance for takeoff [2]. Coupled with increasingly complex integrated mission scenario demand conditions, the shortcomings of conventional fixed-wing UAVs and multi-rotor UAVs are gradually magnified.

In order to make up for the respective shortcomings of fixed-wing and multi-rotor UAVs, UAV scholars have begun the research of combining the advantages of the two types of UAVs, and the research of UAVs in this field has become a popular topic. The existing designs include tilt-rotor, tilt-wing, tail-seat, and composite vertical take-off and landing aircraft [3], among which the tilt-rotor vertical take-off and landing (VTOL) UAVs have attracted much attention from researchers [4,5]. VTOL UAVs combine the vertical lift of helicopters and the speed and range of fixed-wing aircraft, they have a wide range of applications in both military and civil fields [6], and tilt-rotor vertical take-off and landing UAVs have made more progress after a long period of development. Bell completed the development of the “Hawkeye” prototype in 1998. “Hawkeye” adopts the tilt technology of V-22, also loaded with two rotor systems at both ends of the wing, for the tilt-rotor dual rotor UAV, and the rotor power is provided by the Allison 250-C20 turbine engine loaded in the fuselage. The rotors are powered by Allison 250-C20 turbine engines mounted on the fuselage, which can complete a 90° forward tilt in flight. The Korean TR-60 is similar in appearance to the Hawkeye, with a rotary engine in each rotor nacelle to control the propeller tilt, and the power is provided by a drive shaft connected to the engine, with a maximum speed of 250 km/h. In terms of theoretical studies, Huimin Zhao et al. [7] organized the dynamic equations of the UAV into the form of state equations, recorded the constraints on the initial and final values of the control and state variables during the transition process, as well as the constraints on the transition process, and used the Gauss pseudo-spectral method to design an overflight strategy for the optimal control problem with constraints. M.Z. Mimiouni et al. [8] proposed a control surface structure that does not contain any control surface design scheme to develop a control strategy that uses a single controller to handle two flight phases, which optimizes the transition phase performance and enables the UAV to follow any flight trajectory. Chen et al. [9] proposed a leveling-based control assignment method using a Robust Servo Linear Quadratic Regulator (RSLQR) for the control design, which is combined with an expanding state observer to reconfigure the UAV’s unmeasurable state. After this, Ta et al. [10] combined a linear proportional integral differential (PID) controller with a nonlinear saturated S-shaped function to stabilize the pitch angle of a tilt-rotor UAV. Su et al. [11] derived the nonlinear dynamic equations of a tilt-rotor UAV. The model was then linearized around the trimming conditions to generate linear time-invariant state-space models. Adaptive model predictive control (MPC) was used as the controller for each linear model. Chowdhury et al. [12] designed a proportional differential (PD) controller based on backstepping for position and attitude control, and the simulation results proved the stability in hovering experiments and the convergence of the system in tracking experiments. Daniel N. Cardoso et al. [13] proposed a new robust adaptive hybrid controller method for a tilting rotating bi-rotor UAV to handle linear parameter variations. In the literature [14,15], researchers designed a quadcopter that allows its rotors to be tilted around the main axis, and the maneuverability and trajectory tracking capability of the UAV were improved. In the literature [16] the rotor and wing of the vehicle can be tilted together, which reduces the impact caused by wing shading. For the control drive mode of the UAV, most of the current tilt-rotor vertical take-off and landing UAVs use underdriven control schemes; however, the current research on tilt-rotor UAVs is mainly in the area of theoretical analysis and validation, thus lacking prototype experimental validation.

**Figure 1:**
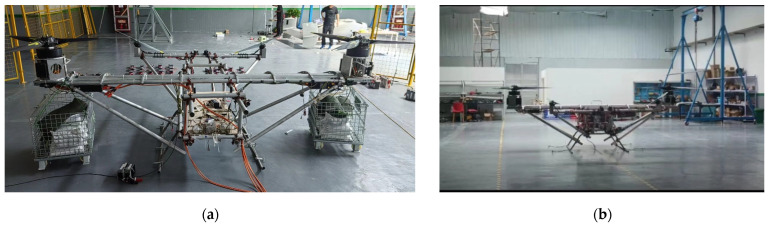
Physical demonstration of the prototype obtained from the final design. (**a**) Prototype in preparation for the experiment. (**b**) Prototype at the start of the experiment.

In this paper, after analyzing the advantages and disadvantages of many kinds of UAVs, based on the lack of experimental prototypes in the field of research on tilt-rotor vertical take-off and landing UAVs, combined with dynamic modeling and analysis, and, at the same time, optimizing the structural control parameters of the experimental machine, the use of vibration characterization reduces and eliminates the hazards of tilt-rotor vertical take-off and landing UAVs when they are in operation. However, the tilt-rotor vertical take-off and landing UAV has more complex characteristics than conventional flights, its flight control has a high degree of sub-linearity and coupling, and there are a large number of uncertain interferences in the flight process. PID control methods have been widely used in engineering practice [17], but the traditional PID control methods have limited anti-interference ability, so the use of a better control method to obtain better control performance of the experimental aircraft is part of the present study. As part of the study, common control methods in the field of UAVs are sliding film control [18], adaptive control [19], model predictive control [20], linear quadratic regulator control [21,22], and serial PID control [23], etc. In order to ensure the rapidity of the control response and the anti-jamming ability of the control system, this study adopts the dual-string PID control to regulate the prototype. Adaptive PID control algorithms currently used in large and complex unmanned aircraft systems still have limitations such as long response time and complex algorithms, whereas the dual-string PID of this study has the advantages of a simple structure and easy implementation while ensuring fast response. In this study, the theoretical analysis of the experimental machine, experimental tests, and vertical takeoff and landing experiments are conducted to collect and calibrate the control data of roll, pitch, and yaw attitude. The final optimization is to obtain the best control effect and control parameter and for the shortcomings of this experiment to put forward future solutions and plans. After optimization and design, the final experimental machine is shown in Figure 1. The ultimate goal of this paper is to make up for the deficiencies of the tilt-rotor vertical takeoff and landing UAV in the experimental field, obtain experimental data, and provide guidance for the research of tilt-rotor vertical takeoff and landing UAV. The main contributions of this paper include the following:

1. Firstly, to meet the requirements of the flight characteristics of a tilt-rotor vertical take-off and landing UAV, a tilt-rotor aircraft with a quad-rotor fuselage layout is introduced and dynamically modeled, and, based on the conclusions of the dynamic modeling, the structure of the experimental aircraft without a skin is reasonably designed.
2. In addition, to meet the landing characteristics and lightweight requirements of vertical take-off and landing UAV, the mechanical characteristics of the fuselage and landing gear are analyzed, and it is proposed to optimize the landing gear structure by adopting the unified objective method and to optimize the main motor seat and the wing ribs of the UAV by using the topology optimization module in workbench.
3. The vibration characteristics of the test aircraft body are analyzed, and a harmonic response analysis method is proposed to be introduced, which, compared with the traditional vibration analysis method, can directly verify and derive the frequency that will produce danger in the actual system, and thus determine the dangerous excitation frequency point for the final tilt-rotor vertical take-off and landing UAV flight.
4. Finally, for the large tilt-rotor vertical take-off and landing UAV, which is subject to many disturbances, long control response time, and complex control algorithms in the working process, this study pioneered the application of dual-serial PID control method to the large tilt-rotor vertical take-off and landing UAV. The control parameters are optimized through simulation, then the simulation and experimental data of the prototype’s flight attitudes, such as roll, pitch, and yaw, are collected, and conclusions are drawn. It provides reference and guidance for the research of tilt-rotor vertical take-off and landing UAVs.

## 2. Complete UAV Design

### 2.1. Dynamic Modeling

**Figure 2:**
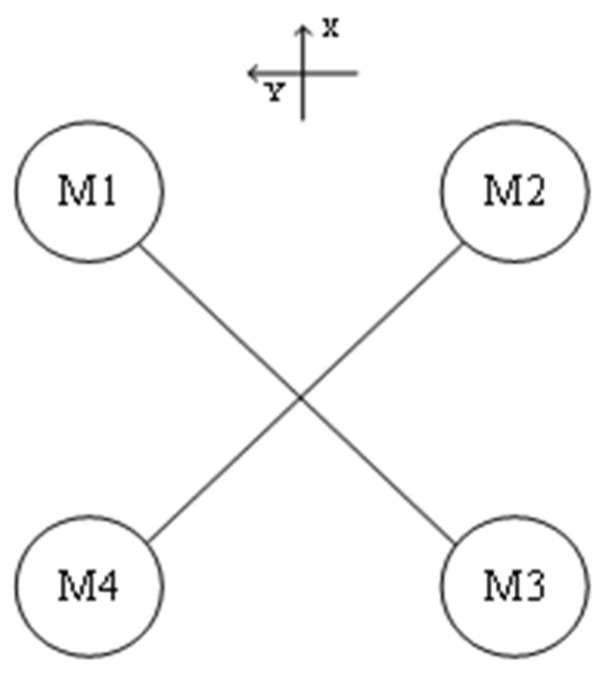
X-mode quad copter drone.

The tilt-rotor UAV of this design is different from the known tilt-rotor UAVs, which is designed with four rotors, and the middle wing is loaded with the main rotor, which provides the main lift for the UAV in the multi-rotor flight mode, and it can be tilted (0~90) degrees during the flight process. Additionally, the tail rotor is a fixed rotor, which cannot be tilted, and it is mainly used to provide the UAV with part of the lift in the multi-rotor flight mode and cooperate with the main rotor to realize the UAV vertical takeoff and landing and flight attitude change. The dynamic model of the tilt-rotor UAV is similar to the “X” quadrotor UAV in flight state, as shown in Figure 2, and the four rotors are matched with different rotational speeds so as to realize the UAV’s four flight states of altitude control, pitch, roll, and yaw.

**Figure 3:**
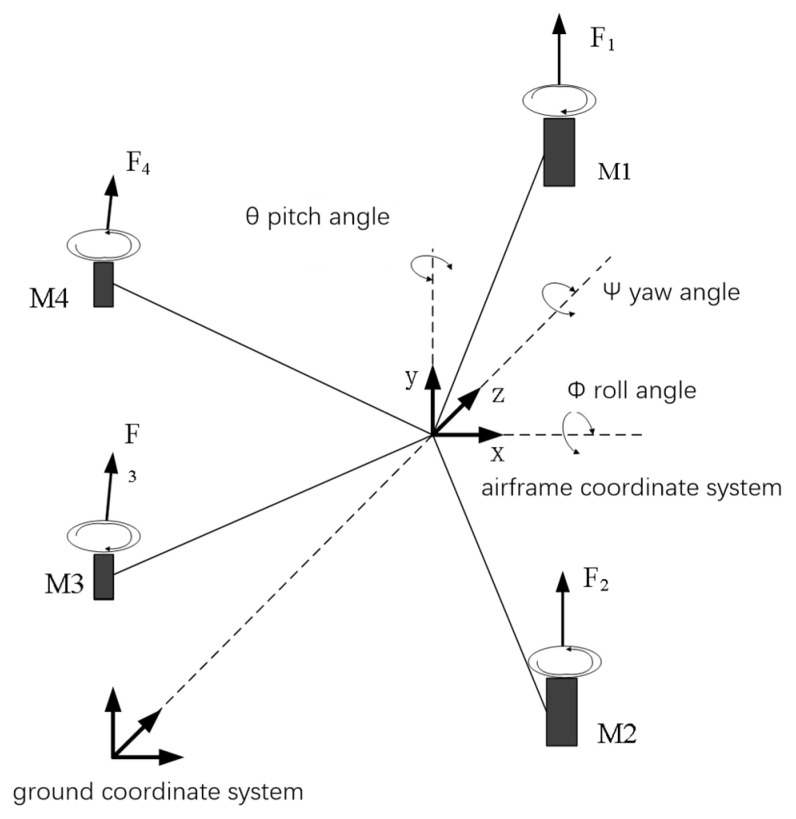
Coordinate system of drone space motion.

The two coordinate systems, ground coordinate system and airframe coordinate system, were established to describe the motion state in the air for the UAV spatial coordinate system. The ground coordinate system was established by choosing an arbitrary position fixed on the earth’s surface as the origin in order to obtain the UAV’s flight information such as attitude heading, and, relative to the takeoff position, the airframe coordinate system is defined on the airframe with the origin of the quadrotor, the Ox-axis is defined in the middle of the left and right end rotor, which makes the left and right end rotor symmetric, the forward flight direction of the UAV is positive, the Oy-axis is defined in the middle of the front and rear end rotor, which makes the front and rear end rotors symmetric, and the Oz-axis passes through the origin perpendicular to the Oxy-plane, as in Figure 3. Again based on this, Euler angles and x, y, z are defined to jointly describe the attitude and position of the UAV, where the Euler angles are the pitch θ, roll ϕ, and yaw ψ angles, respectively. Thus, the overall transformation matrix is obtained [24]:

(1) $$R(\phi ,\theta ,\psi )=\left[\begin{array}{ccc} \cos\theta \cos\psi & \sin\phi \sin\theta \cos\psi -\cos\phi \sin\psi & \cos\phi \sin\theta \cos\psi +\sin\phi \sin\psi \\ \cos\theta \sin\psi & \sin\phi \sin\theta \sin\psi +\cos\phi \cos\psi & \cos\phi \sin\theta \sin\psi -\sin\phi \cos\psi \\ -\sin\theta & \sin\phi \cos\theta & \cos\phi \cos\theta \end{array}\right],$$

Assume that the UAV is a rigid body and does not undergo deformation, that the origin of the camera coordinates of the UAV coincides with the center of mass, that the rotation and revolution of the earth are neglected, and that the takeoff plane is horizontal. The UAV dynamic equation [25] is (4).

(2) $${\vec{F}}_{B}={\vec{F}}_{1}+{\vec{F}}_{2}+{\vec{F}}_{3}+{\vec{F}}_{4},$$

${\vec{F}}_{B}$ is a combined force at the coordinates of the body.

The forces on the UAV in the ground coordinate system are as follows:

(3) $${\tilde{\vec{F}}}_{E}=R(\phi ,\theta ,\psi )\cdot {\tilde{\vec{F}}}_{B},$$

(4) $$\left\{\begin{array}{l} \ddot{x}=\frac{1}{m}\left[\cos\theta \sin\psi \sin\phi \sin\theta \cos\psi -\cos\phi \sin\psi \cos\phi \sin\theta \cos\psi +\sin\phi \sin\psi \right]{\vec{F}}_{B}\\ \ddot{y}=\frac{1}{m}\left[\cos\theta \sin\psi \sin\phi \sin\theta \sin\psi +\cos\phi \cos\psi \cos\phi \sin\theta \sin\psi -\sin\phi \cos\psi \right]{\vec{F}}_{B}\\ \ddot{z}=\frac{1}{m}\left[-\sin\sin\phi \cos\theta \cos\phi \cos\theta \right]{\vec{F}}_{B}-g\\ \ddot{\phi }=\dot{\theta }\dot{\psi }\frac{I_{y}-I_{z}}{I_{x}}+\left({\vec{F}}_{1}\cdot {\vec{l}}_{1}+{\vec{F}}_{4}\cdot {\vec{l}}_{4}-{\vec{F}}_{2}\cdot {\vec{l}}_{2}-{\vec{F}}_{3}\cdot {\vec{l}}_{3}\right)/I_{x}\\ \ddot{\theta }=\dot{\phi }\dot{\psi }\frac{I_{z}-I_{x}}{I_{y}}+\left({\vec{F}}_{1}\cdot {\vec{l}}_{1}+{\vec{F}}_{2}\cdot {\vec{l}}_{2}-{\vec{F}}_{3}\cdot {\vec{l}}_{3}-{\vec{F}}_{4}\cdot {\vec{l}}_{4}\right)/I_{y}\\ \dot{\psi }=\dot{\theta }\dot{\phi }\frac{I_{x}-I_{y}}{I_{z}}+\left(d_{1}\cdot {\vec{F}}_{1}+d_{3}\cdot {\vec{F}}_{3}-d_{2}\cdot {\vec{F}}_{2}-d_{4}\cdot {\vec{F}}_{4}\right)/I_{z} \end{array}\right.,$$

${\vec{F}}_{1}$, ${\vec{F}}_{2}$, ${\vec{F}}_{3}$, and ${\vec{F}}_{4}$ are the lift generated by the rotor; $\ddot{x}$, $\ddot{y}$, and $\ddot{z}$ correspond to the acceleration on the three coordinate axes; m is the mass of the UAV; $I_{x}$, $I_{y}$, and $I_{z}$ are the inertia of the air frame; ${\vec{l}}_{1}$, ${\vec{l}}_{2}$, ${\vec{l}}_{3}$, and ${\vec{l}}_{4}$ are the force arm from the center of the rotor to the origin of the air-frame coordinate system; $d_{1}$, $d_{2}$, $d_{3}$, and $d_{4}$ are the performance parameter of the propeller.

It can be found through the dynamic model that the pitch, roll, and yaw attitude control of the tilt-rotor concept UAV in multi-rotor mode is related to its mass, rotational inertia, force arm from the rotor to the center of gravity, and lift force generated by the power system. Among them, the ones that have a greater influence on the UAV state in the operational state are the pulling force generated by the rotor and the power system, and the pulling force and power of the rotor will be investigated below.

**Figure 4:**
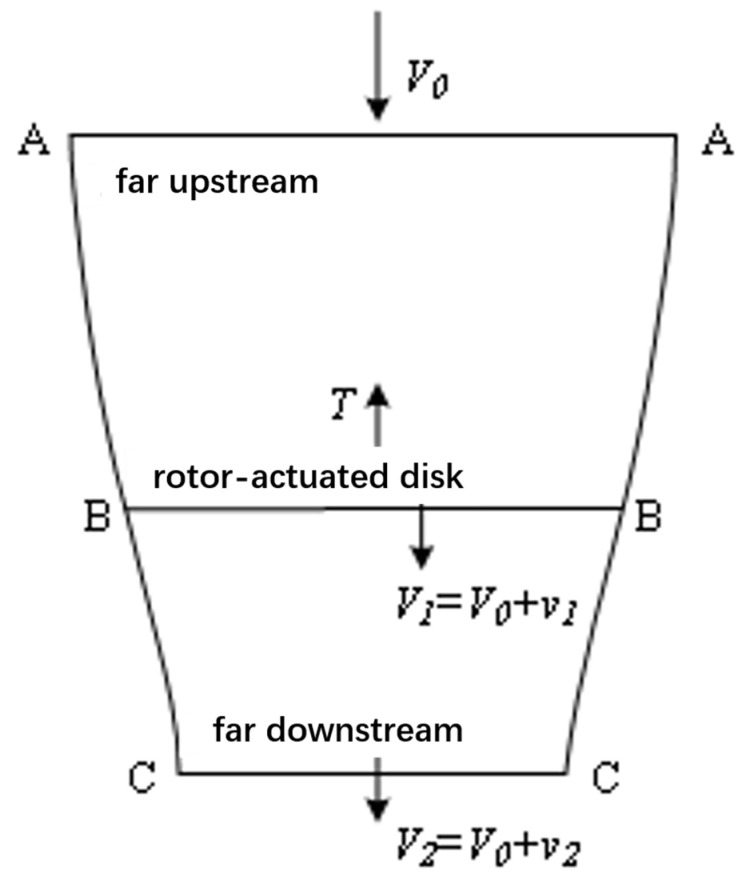
Rotor action disk model of momentum theory in vertical rise state.

Combined with the rotor momentum theory to analyze the generation and change in airflow, as shown in Figure 4, assuming that the air is an ideal fluid, the rotor is infinitely thin and uniformly in contact with the air, and the airflow is constant at each point of the action disk. To establish a uniform load rotor disk model as shown in Figure 4, B-B cross-section is the rotor disk, A-A cross-section and C-C cross-section are the upstream and downstream boundaries, $V_{0}$ is the velocity of the airflow in the 0-0 cross-section, the velocity of the airflow at the B-B cross-section is $V_{1}$, the velocity of the airflow reaches the C-C cross-section, the velocity increases to $V_{2}$, and $v_{1}$ and $v_{2}$ are the induced velocity of the airflow at the two cross-sections. The following relation is obtained based on the theorem of momentum, the theorem of conservation of mass, and the theorem of conservation of energy:

(5) $$T=T^{\prime }=m(V_{2}-V_{0})=mv_{2},$$

$T^{\prime }$ is the external force acting on the fluid by the rotor, T is the rotor tension; m is the fluid mass; $V_{0}$, $V_{2}$ are the airflow velocity; and $v_{2}$ is the local airflow-induced velocity far downstream.

(6) $$m=\rho V_{0}S_{0}=\rho V_{1}S_{1}=\rho V_{2}S_{2}=\mathrm{constant},$$

ρ is the air density; $S_{0}$, $S_{1}$, and $S_{2}$ are the upstream distant, action disk, and downstream distant areas, respectively.

The power consumed by the rotor is determined by the rate of change in kinetic energy of the fluid within the model:

(7) $$P=m(V_{2}^{2}-V_{0}^{2})/2=m(V_{0}+v_{2}/2)v_{2},$$

Since the rotor power is equal to the product of the rotor pull force and the airflow velocity $V_{1}$ at the rotor action disk, expressed as follows:

(8) $$P=TV_{1}=TV_{0}+Tv_{1},$$

At the rotor action disk, $V_{1}=V_{0}+v_{1}$, $S=\pi R^{2}$, which ultimately gives the relationship between rotor pull and power:

(9) $$T=2\rho \pi R^{2}\left(V_{0}-v_{1}\right)v_{1},$$

(10) $$P=2\rho \pi R^{2}{\left(V_{0}-v_{1}\right)}^{2}v_{1},$$

It is known through the rotor momentum theory that, in the multi-rotor mode, the power and lift of the rotor are only related to the local induced velocity and the area of the propeller disk. The above theoretical analysis was carried out in the case of no skin, while the reality of the tilt-rotor UAV exists in the skin, when the body exists in the skin, and due to the wing being in the rotor directly under the rotor, there will be a certain impact on the rotor propeller area, compared to the case of no skin in which there will be a certain amount of power and tension loss. This situation can be solved by the rotor power and rotational speed in the selection of the power system. When selecting the power system, we need to pay attention to this effect. Other than that, the wing skin does not affect the rotor blades in any other way. Therefore, in order to reduce the weight of the fuselage and increase the endurance of the tilt-rotor UAV, combined with the dynamic model of the multi-rotor mode, in this study, without considering the fuselage skin, and ensuring that the weight of the prototype and weight distribution, structural form and power system are the same as that of the tilt rotor concept UAV, the prototype can be used to carry out the vertical takeoff and landing test, so as to provide the test data and guidance for the research of the tilt-rotor concept UAV.

### 2.2. Fuselage Design

The UAV studied in this paper needs to adapt to two working conditions. Working condition one is the ground test of the whole aircraft, mainly testing the remote communication function of the control system and the performance of the power system. Working condition two is to complete the vertical takeoff and landing of the test aircraft and hovering process of pitch, roll, yaw, and other attitude controls. Taking into account the cost and process characteristics, and at the same time need to meet the mechanical and dynamic performance indicators, the design of the design indicators is shown in Table 1.

**Figure 5:**
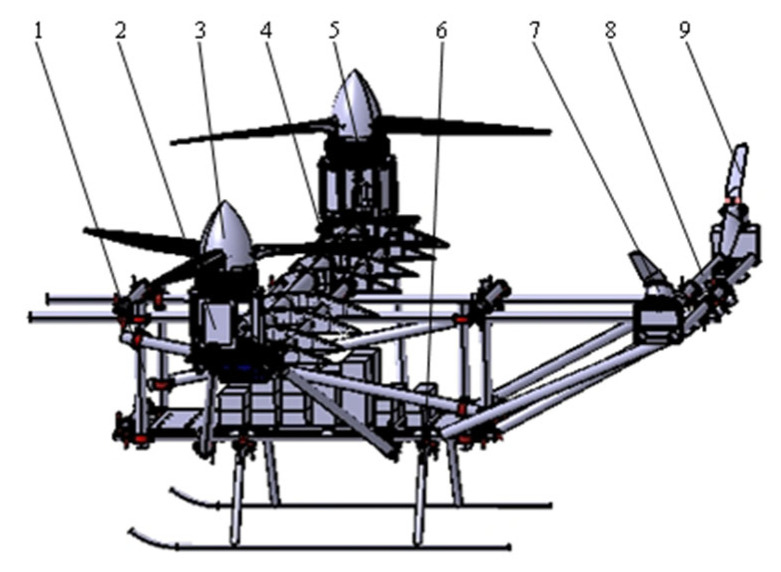
Diagram of the overall structure of the UAV without considering the skins. 1, fuselage; 2, nacelle; 3, main propeller; 4, wing; 5, main motor; 6, landing gear; 7, tail motor; 8, tail wing; and 9, tail propeller.

According to the requirements, the material selection of the prototype should ensure that the structural strength and stiffness of the basis achieve the minimum mass, but it also needs to reduce the cost of the experiment. After comprehensive consideration of material density, tensile modulus, tensile strength, specific modulus, specific strength, and other performance indicators, it was decided to use aluminum alloy and titanium alloy UAV body structure materials. The airframe design requires reasonable layout structure, lightweight, easy disassembly and maintenance, safety, and economy. Therefore, the overall structure of this design is shown in Figure 5. The test aircraft wing and tail wing were connected to the upper fuselage through cross fasteners, which were distributed and installed at 1015 mm and 2809 mm from the front end of the fuselage, and the four rotors were installed at the ends of the wing and the tail wing, respectively. The wing parameters refer to the cruise lift coefficient of the same class of fixed-wing aircraft $C_{Lwing}$ (0.8) to obtain the wing area of about 3 m^2^ and refer to the twin-engine airplane, which has the following characteristics: the wing chord ratio AR is 12, the $C_{ave}$ is about 0.5 m, the wing length is 6 m, the weight of the experimental test aircraft is large, the wing bears the main propeller tension during the flight, the three-beam structure can satisfy the requirements, the main beam has sufficiently high strength, the aileron strength is weaker, and according to the structural requirements and the characteristics of the wing, the main beam is located at 26% of the wing chord, and the two aileron beams are located at 8% and 57% of the wing chord, respectively.

(11) $$S_{wing}=\frac{Mg}{\rho VC_{Lwing}},$$

$S_{wing}$ is the wing area; M is the whole aircraft design mass; and V is the design flight speed.

(12) $$C_{ave}=\sqrt{\frac{S_{wing}}{AR}},$$

(13) $$B=AR\times C_{wing},$$

B is the spread length.

Tail parameter reference weight leveling, using Formulas (11), (12), and (14), calculated flat tail area of about 1.0976 m^2^. Tail structure design to meet the strength and stiffness requirements can be, at the same time, to leave enough space for the electronic speed controller. Therefore, the tail structure form uses double-beam type, and the two beams $l_{h}$ have the same structural parameters, are used in a length of 2260 mm, inner diameter of 44 mm, outer diameter of 48 mm, 6061 aluminum alloy tube, and tail two-beam spacing of 140 mm.

(14) $$V_{ht}=\frac{l_{h}S_{h}}{C_{ave}S_{wing}},$$

$l_{h}$ is the length from the quarter chord position of the flat tail to the center of gravity; $S_{h}$ is the area of the flat tail; and $V_{ht}$ is the capacity of the flat tail.

The fuselage design takes into account that this experimental aircraft is only used for vertical takeoff and landing and hovering flight, and it is required to be easily disassembled and transported on the basis of cost reduction, so it was selected to be of ko-truss-beam structure, the landing gear was selected to be the common sled-type landing gear, and the bow beam and the left and right sled of the landing gear were welded together with the titanium alloy round tubes with the inner diameter of 26 mm and the outer diameter of 30 mm, and the height of the sled-type landing gear was designed to be 385 mm. The height of the skid landing gear was designed to be 385 mm, the spacing between skids was designed to be 1166 mm, and the spacing between the front and rear bow beams was initially designed to be 800 mm.

## 3. Mechanical Characterization and Structural Optimization of Prototype

### 3.1. Static Analysis

The strength and stability of the body structure of the prototype are crucial to the performance of the prototype. The designed model of the prototype body is imported into the analysis software; the prototype fuselage, wings, tail, and other related structures are the main load-bearing components of the prototype, which need to be retained; and the bearings, bolts, and other parts of the overall impact of the prototype are relatively small and appropriately simplified. The designed three-dimensional model of the prototype body is imported into Workbench, and tetrahedral meshing is used for the parts with a complex structure and small size. Hexahedral meshing is used for the structural rules, and the mesh density of the main load-bearing parts is increased appropriately.

According to Table 1, the maximum design weight of the prototype is 350 kg, the design weight of the fuselage is 34.9 kg, the design weight of the wing is 33.5 kg, the design weight of the tail is 6.9 kg, the design weight of the wing powertrain is 117.3 kg, the design weight of the tail powertrain is 20.4 kg, and the design weight of the onboard equipment and the landing gear is 137 kg. The fuselage, wing, and tailplane are made of 6061 aluminum, the connecting fasteners are made of ductile cast iron, and the main motor mount connectors are made of structural steel. The main rotor can provide a maximum lift of 1800 N unilaterally, and the tail rotor can provide a maximum lift of 500 N unilaterally. The powertrain, airborne equipment, and landing gear are simplified to be given to the fuselage in the form of force. Taking the safety factor of 1.25, the lift force is 2250 N distributed on both sides of the wing and 625 N distributed on both sides of the tail rotor.

**Figure 6:**
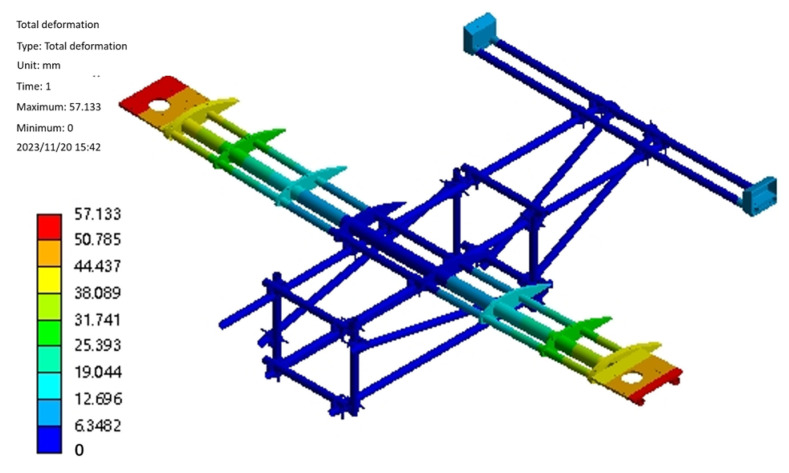
Deformation of the body under force.

**Figure 7:**
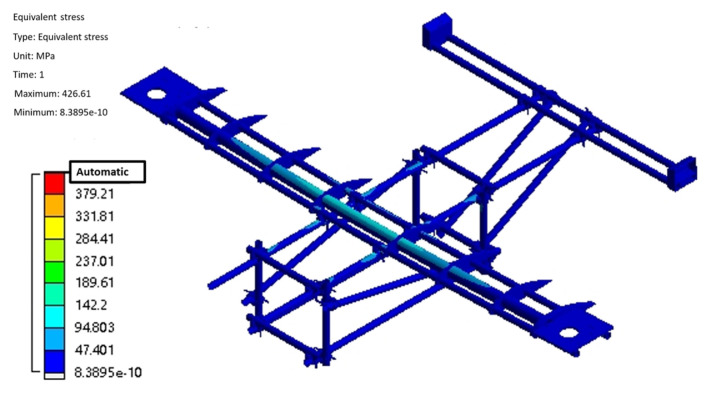
Stresses on the body.

The flight process of the test aircraft has six degrees of freedom, and the constraints are applied by the method of inertial release, assuming it is stationary, to complete the hydrostatic analysis. After the boundary conditions are applied, the static solution of the test aircraft is started, and the results are shown in Figure 6 and Figure 7. From Figure 6, it can be seen that the maximum deformation of the test aircraft occurs at the edge of the wing beam and the main motor seat after the force is applied. Also, the maximum deformation value is 57.1 mm, and the ratio of its half wingspan is about 2.82%, which meets the design requirement of less than 3%, so the stiffness of the test aircraft meets the design requirement. As can be seen from Figure 7, the maximum equivalent force of the structure is concentrated in the scaffolding fasteners, and the maximum equivalent force value is 379.21 MPa, which is smaller than its minimum strength limit of 450 MPa; the maximum stress concentrated in the aluminum alloy tubes of 6061 and 7075 is 142.2 MPa, which is smaller than its minimum ultimate strength of 240 MPa and 455 MPa, so the structural strength of the prototype meets the design requirements. The total weight of the test aircraft body structure with wings and tail is about 75.3 kg, accounting for 21.5% of the maximum design weight, which is close to the design requirements, and part of the structure will be optimized subsequently, which can further reduce the total weight of the structure.

### 3.2. Mechanical Properties Analysis of Landing Gear

#### 3.2.1. Landing Gear Static Analysis

**Figure 8:**
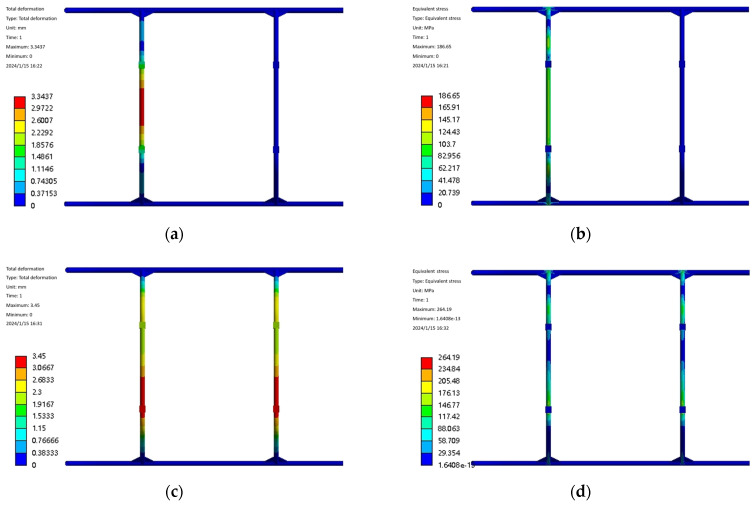
Static force analysis of two working conditions of landing gear. (**a**) Deformation cloud map for working condition 1. (**b**) Stress map for working condition 1. (**c**) Deformation cloud map for working condition 2. (**d**) Stress map for working condition 2.

Landing gear static mechanical analysis of landing gear in the prototype for smooth lifting and lowering plays an important role. The structural design of the landing gear is to ensure that the structural strength and rigidity of the premise maintain the quality of light. The landing gear designed in this paper is expected to have a certain elastic deformation capacity to absorb the impact of the kinetic energy of the landing of the test aircraft, but it is also expected to have enough strength to avoid fracture due to excessive deformation. In the process of designing the landing gear, it is necessary to rationalize the design by combining the UAV design, strength, and stiffness requirements. The landing gear model is imported into Workbench for meshing, then the boundary conditions are set, and the load is applied. The landing gear is mainly subject to the extreme impact of the experimental aircraft when it lands, and, according to the landing situation, the landing gear is subjected to the load in two working conditions: working condition one is that, when the experimental aircraft lands with a left tilt or a right tilt, the landing gear is subjected to the main forces on both sides of the gear, and the safety coefficient is taken to be 1.5, with an average force of 2625 N on the left and right two connecting points. The average force is 2625 N. In Case 2, when the experimental aircraft is tilted low or tilted back, the front and rear ends of the landing gear are subjected to the main force, and the average force at the front and rear connection points is 2625 N. The results of this analysis are shown in Figure 8. From the stress–strain diagrams, it can be seen that, in Case 1, the front and rear bow beams are subjected to the force individually, the deformation is mainly concentrated in the middle of the bow beam, and the stress is concentrated on the both sides of the stress point of the bow beams and at the connection point between the bow beam and the skid. The deformation and stress are concentrated in the middle of the single arch beam, and the stress is concentrated on both sides of the stress point of the single arch beam and the connection between the arch beam and the skid. The deformation and equivalent stresses of the landing gear under two different working conditions are shown in Table 2, from which it can be seen that the maximum deformation and equivalent stresses of the landing gear under working condition 1 are small, while the maximum deformation and equivalent stresses under working condition 2 are large, and the static analysis of the landing gear under the two working conditions meets the requirements of the yield strength, so that the landing gear structure meets the design requirements.

#### 3.2.2. Simulation Analysis of Landing Gear Impact

**Figure 9:**
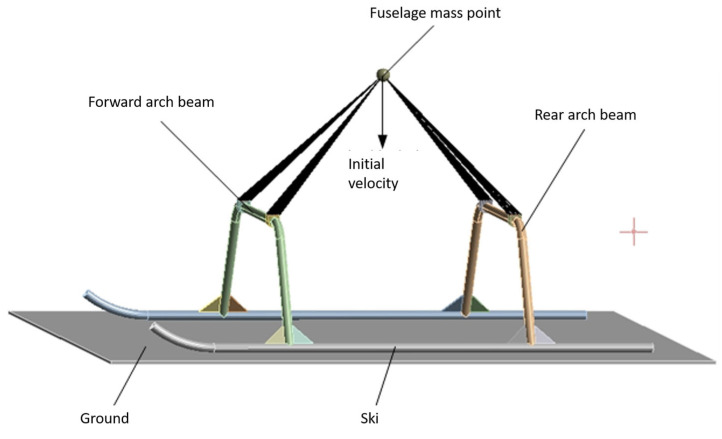
Finite element model of landing impact of skid landing gear.

Since the fuselage structure is very complex, there are problems such as the difficulty of mesh division and too much increase in simulation calculation time, and the fuselage hardly produces energy-absorbing deformation during the landing impact of the test aircraft, so taking into account the efficiency of the simulation calculations, the fuselage mechanism is not taken into account when analyzing the landing impact of the landing gear, and the mass points are used to represent it. The mass point is set at the center of gravity of the fuselage, the four connection points of the landing gear bow girder and the fuselage are used as the mass point connection locations, the constraints are set as rigid body connections, the attributes of the mass point are set as rigid, the mass and rotational inertia of the fuselage are assigned to the mass point, and the impacted ground is simulated by a rigid wall. The landing gear finite element model is shown in Figure 9.

**Figure 10:**
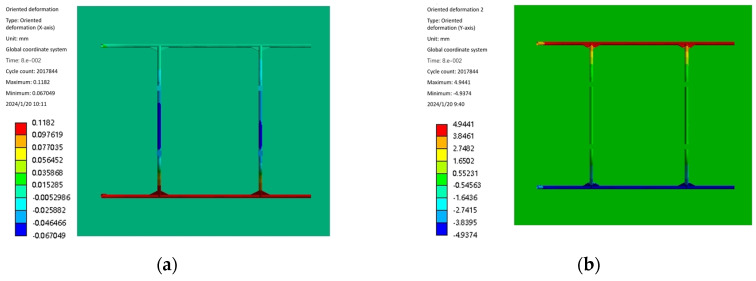

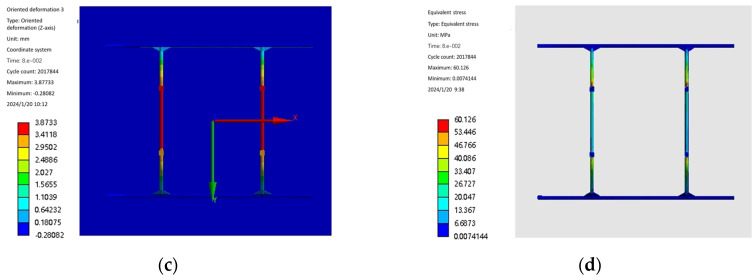
Simulation cloud diagram of landing gear impact for Case 1. (**a**) The 0.15 m/s directional deformation x-axis cloud map. (**b**) The 0.15 m/s directional deformation y-axis cloud map. (**c**) The 0.15 m/s directional deformation z-axis cloud map. (**d**) The 0.15 m/s equivalent force map.

**Figure 11:**
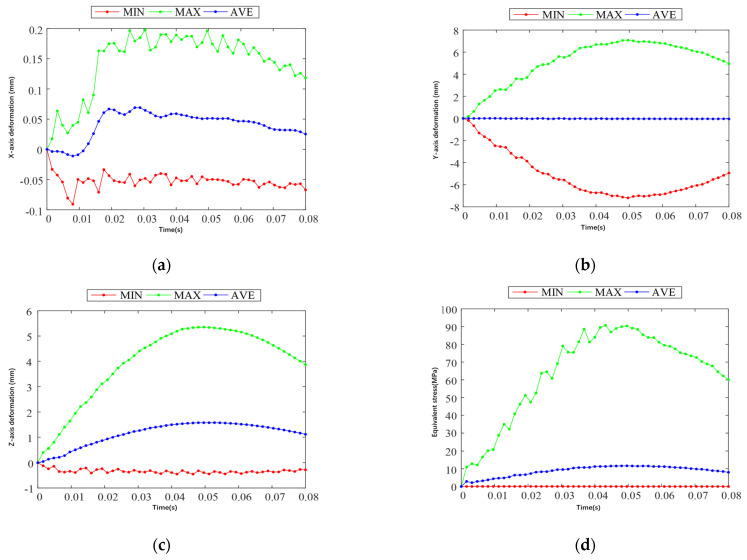
Simulation curve of landing gear impact for condition 1. (**a**) X-direction deformation. (**b**) Y-direction deformation. (**c**) Z-direction deformation. (**d**) Equivalence change.

The time used in the simulation is set to 0.08 s, the constraint mode of the skid and the ground model is set to friction, and the friction coefficient is set to 0.15. Relevant studies have proved with the statistical results of helicopter landing sinking speed test data per 1000 times that the speeds that appear more frequently are 0.15 m/s, 0.45 m/s, 0.76 m/s, 1.07 m/s, and 1.37 m/s. Also, the landing speed is selected to appear the most times in the actual aircraft of 0.15 m/s, combined with a maximum speed of 1.37 m/s, to conduct the comparison and analysis, and to select the appropriate landing speed. The deformation and stress of the landing gear are tested in two separate cases. When the test aircraft landed at a speed of 0.15 m/s, Figure 10 shows the results of the landing gear landing impact simulation and analysis of cloud diagrams, respectively, along the x, y, and z-axis direction of the deformation of the cloud diagram and the equivalent force cloud diagram. Figure 11 shows the deformation and equivalent force change curve along the x, y, and z directions during the landing gear working condition of one impact landing.

From Figure 10a, it can be seen that there is almost no deformation of the landing gear along the x-axis direction during the landing impact in working condition 1, which is in line with the actual working condition. Figure 10b shows that the landing gear is symmetrically deformed along the y-axis during the landing impact, which is mainly caused by the deformation of the skid and the connection between the bow beam and the skid and the sled, which is caused by the sliding of the skid along the y-axis. Figure 10c shows that, along the z-axis, the deformation is mainly generated by the bow beam, and the deformation is concentrated in the middle position of the bow beam during the landing impact of the landing gear. Figure 10d shows that the middle effect force during the landing impact of the landing gear is mainly concentrated in the connection between the bow-shaped beam and the UAV and the bending of the bow-shaped beam.

**Figure 12:**
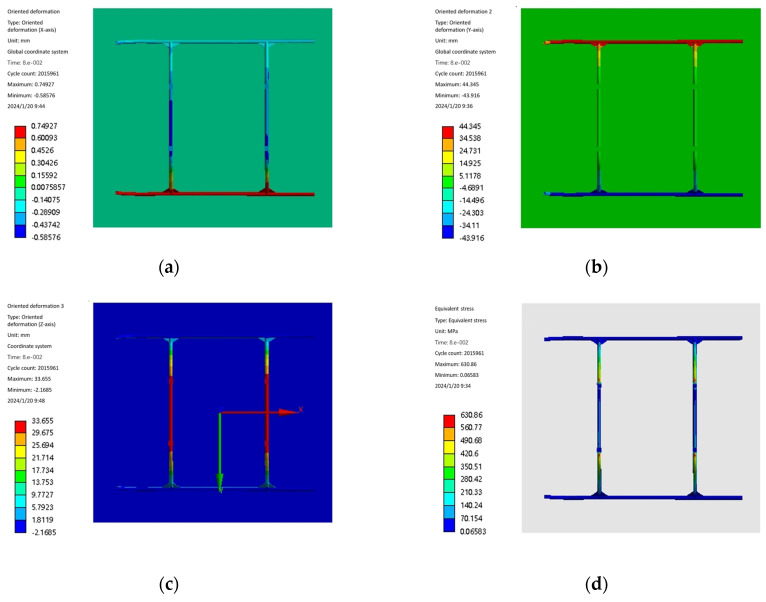
Simulation cloud diagram of landing gear impact in Case 2. (**a**) The 1.37 m/s directional deformation x-axis cloud map. (**b**) The 1.37 m/s directional deformation y-axis cloud map. (**c**) The 1.37 m/s directional deformation x-axis cloud map. (**d**) The 1.37 m/s equivalent force map.

From Figure 11b, it can be seen that the deformation of the landing gear along the y-axis direction reaches the maximum value of 7.08 mm in 0.0496 s during the landing impact; from Figure 11c, it can be seen that the deformation of the landing gear along the z-axis direction reaches the maximum value of 5.35 mm in 0.0496 s during the landing impact; and from Figure 11d, it can be seen that the equivalent force of the landing gear reaches the maximum value of 90.38 MPa in 0.0496 s during the landing impact. Both deformation and equivalent force in the y-axis and z-axis directions reach the maximum value of 90.38 MPa after maximum displacement and maximum equivalent force. Figure 12d shows that, during the landing impact of the landing gear, the equivalent force reaches the maximum value of 90.38 MPa in 0.0496 s. The deformation and equivalent force of the landing gear in the direction of the y-axis and z-axis reach the maximum value in 0.0496 s, and the landing gear starts to rebound upward after the maximum displacement and maximum equivalent force, which is in line with the actual impact law of the landing gear.

The simulation cloud diagram when the prototype landed at maximum speed is shown in Figure 12.

**Figure 13:**
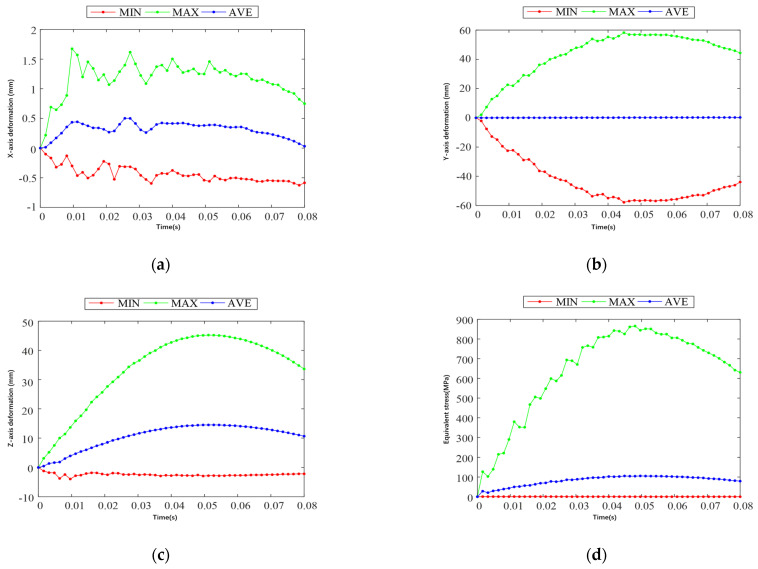
Simulation curve of landing gear impact for working condition 2. (**a**) X-direction deformation. (**b**) Y-direction deformation. (**c**) Z-direction deformation. (**d**) equivalence change.

The simulation cloud diagram of landing gear impact in Case 2 shows that the main deformation areas along the x-axis, y-axis, and z-direction during landing impact in Case 2 are consistent with the landing impact in Case 1, and the distribution of equivalent force is also similar. Figure 13 shows the deformation and equivalent force change curves along the x, y, and z directions during the landing impact of the landing gear in Case II. From Figure 13b, it can be seen that the deformation of the landing gear along the y-axis direction in the landing impact process reaches the maximum at 0.0576 s, and the maximum value is 56.80 mm; from Figure 13c, it can be seen that the deformation of the landing gear along the z-axis direction in the landing impact process reaches the maximum at 0.0576 s, and the maximum value is 44.62 mm; and Figure 13d shows that the medium effect force in the landing gear landing impact process reaches its maximum at 0.0576 s, with a maximum value of 824.88 MPa. The landing gear deformation in the y-axis direction, the z-axis direction, and the equivalent force reach the maximum value in 0.0576 s. After the maximum displacement and the maximum equivalent force, the landing gear starts to rebound upward.

When the landing gear is in a 0.15 m/s speed impact landing, the maximum stress of 90.38 MPa is far less than the yield strength of the landing gear, 825 MPa. When the landing gear is in a 1.37 m/s speed impact landing, the maximum stress of 824.88 MPa is close to the yield strength of the landing gear. The actual flight should be avoided in a 1.37 m/s speed landing situation. In order to control the landing gear, the safety coefficient can reach more than 1.3, so the speed of the test aircraft is controlled between 0.15 m/s and 1 m/s, which is able to realize a safe landing.

### 3.3. Optimization of Tester Body Structure

#### 3.3.1. Landing Gear Structure Optimization

Multiple variables are involved in the optimization of the landing gear structure [26], so the unified objective method is introduced to iteratively optimize the beam length L and pinch angle α in the landing gear. Assuming that there are i design variables and j objective functions, the multi-objective function of the model can be expressed as follows:

(15) $$\left\{\begin{array}{l} \begin{array}{l} \min F(X)=\min{\left[f_{1}(X),f_{2}(X),\cdots ,f_{j}(X)\right]}^{T}\\ s.t.\left\{\begin{array}{l} g_{u}(X)\leq 0,(u=1,2,\cdots ,m)\\ h_{u}(X)\leq 0,(u=1,2,\cdots ,n) \end{array}\right. \end{array}\\ X=[x_{1},x_{2},\cdots ,x_{i}] \end{array}\right.,$$

F(X) is the objective function; $g_{u}$ and $h_{u}$ are the constraint functions; and X is the target variable.

Combining the linear weighting method to form a composite objective function from multiple objective functions, a function F(X) to be minimized is specified as a union of the properties in question to obtain the composite objective function:

(16) $$F\left(X\right)=W_{1}\cdot f_{1}\left(X\right)+W_{2}\cdot f_{2}\left(X\right)+\cdots +W_{r}\cdot f_{s}\left(X\right),$$

$W_{r}$ is the weighting factor. It is used to indicate the importance of each objective function with the following relationship:

(17) $$\left\{\begin{array}{l} W_{r}>0\\ \sum_{r=1}^{s}W_{r}=1,(k=1,2,\cdots ,s) \end{array}\right.,$$

**Figure 14:**
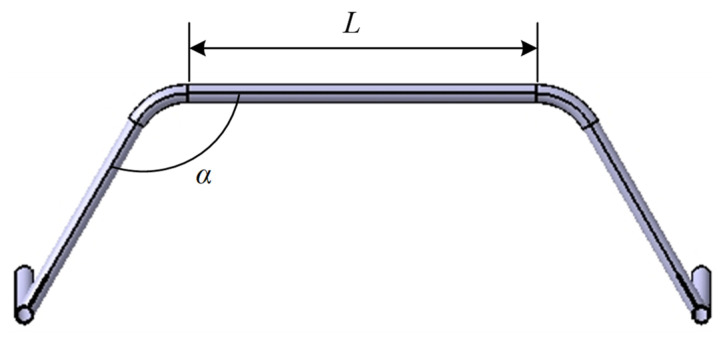
Schematic diagram of design variables.

When optimizing the design variables L and α, the target variables are chosen as the maximum equivalent force σ and the landing gear mass m occurring in this work. As shown in Figure 14, theoretically there exists a certain geometrical relationship between L and α, and α will follow to produce a change when L is changed, but due to the existence of a chamfering angle, it can be made so that α does not have to change. Therefore, in a certain range, there exists a certain combination of relationship between L and α, which together affects the structure of the landing gear. By testing the geometric relationship in the 3D model, the range values of L and α can be obtained separately.

Then the mathematical model of landing gear optimization design can be expressed as follows:

(18) $$\begin{array}{l} \min F(X)={\min[m(X),\sigma (X)]}^{T}\\ X={\left[L,\alpha \right]}^{T}\\ 550\leq L\leq 600,115{}^{\circ}\leq \alpha \leq 125{}^{\circ}\\ F(X)=W_{1}\cdot m(X)+W_{2}\cdot \sigma (X) \end{array},$$

Considering that the maximum stress indicator needs to be prioritized, $W_{1}=0.3,W_{2}=0.7$.

The mathematical model with accurate parameters is obtained, the input parameters of this optimization are the landing gear bowtie beam structural variables, rod length L and angle α, and the output variables are the minimum mass of the landing gear and the maximum equivalent force in the simulation analysis. The sensitivity analysis of the input and output parameters is carried out, and it is found that the rod length L of the bowtie beam is positively correlated with both the maximum equivalent force and minimum mass, and the angle α of the bowtie beam is negatively correlated with both the maximum equivalent force and the minimum mass. Then the landing gear optimization design is realized through the response surface optimization module in Workbench 2023 R2, SolidWorks 2023 and Workbench 2023 R2 interacted, the parameterization of the design variables is carried out in SolidWorks, after that the analysis is carried out in finite element software, and the landing gear landing speed is taken as 1 m/s. Finally, the response surface module will collect the sample points through the intermediate material method. The optimization is performed by fitting the samples.

**Figure 15:**
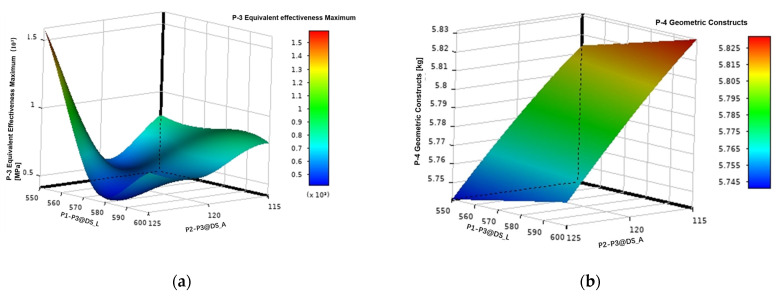
Schematic of the parametric response surface. (**a**) Maximum equivalent force (physics). (**b**) Minimum mass.

The response surface shown in Figure 15 is obtained by polynomial fitting the input and output parameters, where DS_L represents the arch beam rod length L, and DS_A represents the angle α of the arch beam. It can be seen from the figure that, in the range of the two parameters, the maximum equivalent force σ is not completely linear with the input parameters, and the two parameters jointly influence the change in the maximum equivalent force, while the minimum mass is almost linear with the two parameters, and the mass m increases with the increase in the rod length L and decreases with the increase in the angle α.

This optimization has two purposes: on the one hand, it is to reduce the weight of the landing gear to ensure that the overall design weight of the prototype does not exceed the maximum value required by the design, and on the other hand, it is to reduce the maximum stress value during the operation of the landing gear and improve the strength of the landing gear. After the optimization of the response surface dimensions, the optimization results are shown in Table 3. The optimization of the landing gear mass reduction is not obvious, so the smallest equivalent stress value is selected as the optimal result in the optimization data. Finally, the length of the bow beam of the landing gear is reduced to 579.15 mm, the angle of the bow beam is increased to 123.9°, and the overall mass of the landing gear is reduced by 0.037 kg, which is not significant, but the static strength is greatly increased. The maximum equivalent force of the optimized landing gear is calculated by the method of calculating the static strength, and under the same conditions, the maximum equivalent force is reduced by 204.08 MPa, but it is still necessary to simulate the equivalent force of the landing gear in the real scenario through subsequent experiments.

**Figure 16:**
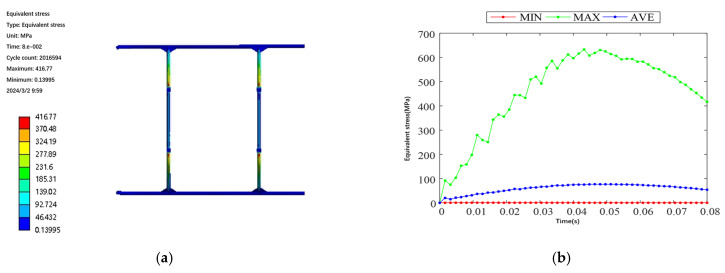

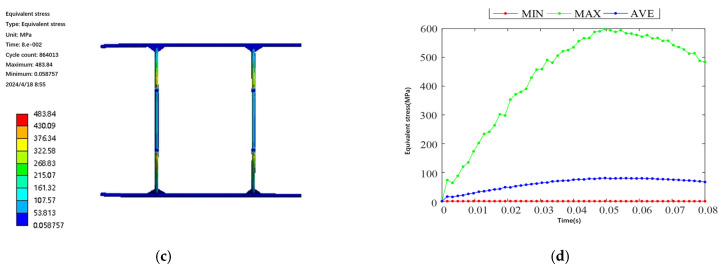
Simulation of landing gear impact at 1 m/s speed after optimization. (**a**) The 1 m/s equivalent force cloud before optimization. (**b**) The 1 m/s equivalent force curve before optimization. (**c**) The 1 m/s equivalent force cloud after optimization. (**d**) The 1 m/s equivalent force curve after optimization.

The landing impact simulation of the landing gear before optimization at a speed of 1 m/s is shown in Figure 16a,b, and it is found that the maximum stress of the landing gear is 630.74 MPa. The landing gear will be redesigned according to the optimized size and imported into the display dynamic analysis software to re-test the landing impact simulation. Simulation constraints as well as boundary conditions remain unchanged, the same selection of the landing gear is set to 1 m/s speed for impact calculations, the end of the computation time is set to 0.08 s, to obtain the optimized 1 m/s equivalent force cloud and the equivalent force curves as shown in Figure 16c,d.

From the optimized landing gear impact simulation cloud and curve diagrams shown in Figure 16c,d, it can be seen that the place where the maximum stress of the landing gear occurs is still at the bow-beam bending corner, and the equivalent stress has the same trend as that before optimization, and the maximum stress is 598.11 MPa, which is reduced by 32.63 MPa compared with that before optimization.

#### 3.3.2. Wing Ribs and Main Motor Mount Optimization

**Figure 17:**
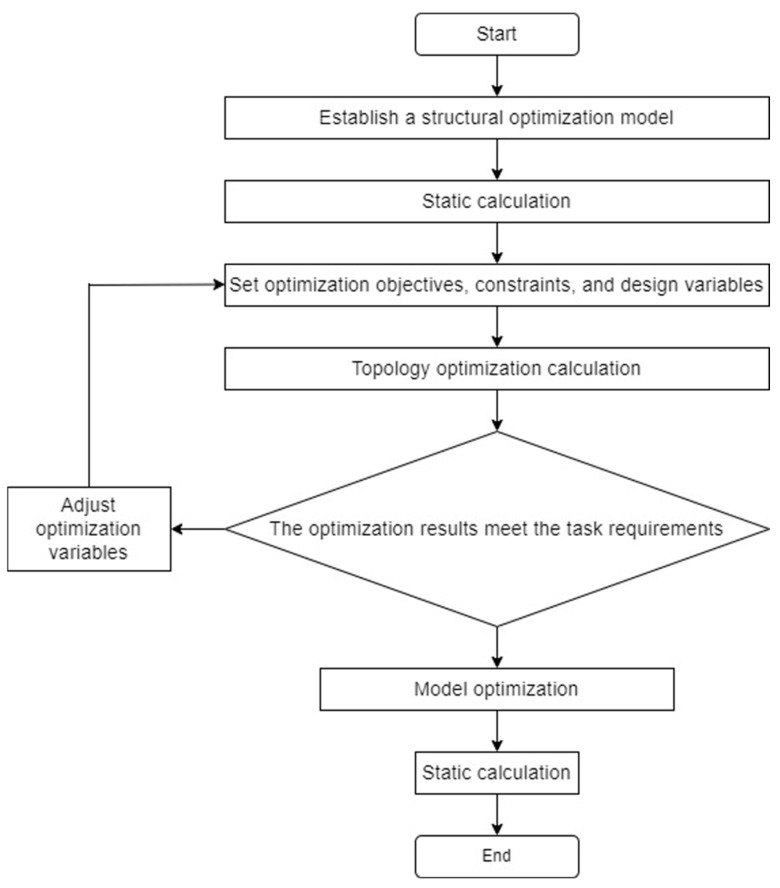
Topology optimization flow.

The wing ribs and the main motor seat are optimized using the topology optimization method. Combining the variable density method of the topology optimization module in Workbench and the static structure module together to complete the optimization, after importing the 3D models of the main motor seat and the wing ribs into Workbench, the optimization setup is carried out, and the optimization process is shown in Figure 17.

According to the actual selection of materials, the structural material properties are setup and model meshing. In order to ensure the accuracy of the calculation results, the average mesh quality needs to be greater than 0.7, so the mesh size is selected as 5 mm, locally encrypted to 1 mm. The topology optimization region will be divided into the force transfer structure, and bolts and other mounting holes will be divided into non-optimized areas, according to experience. To retain the structural reinforcement, the rest of the structure can be divided into optimized areas to achieve a better optimization effect. According to the experience, the structural reinforcement is retained, and the rest of the structure can be divided into optimized regions to achieve a better optimization effect. Setting the mass percentage as the reference value of constraints, in order to obtain better optimization results, the reference value of motor seat topology optimization is 0.6, and the reference value of wing rib topology optimization is 0.55. The prototype designed in this paper has a total of 8 wing ribs, of which 2 are reinforcing ribs, and the rest are ordinary ribs. The reinforcing ribs are the main load-bearing components, and the common ribs mainly play the role of stabilizing the main beam of the wing, so the topology optimization of the wing ribs in this section mainly optimizes the reinforcing ribs, and the rest of the wing ribs refer to the optimization results of the reinforcing ribs.

**Figure 18:**
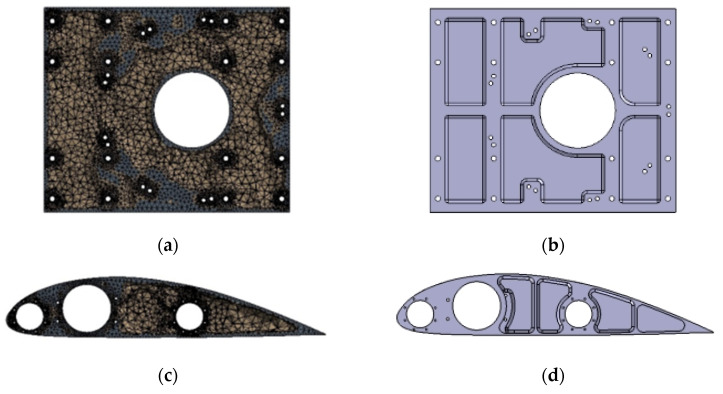
Optimization of structural components. (**a**) Motor mount after topology optimization. (**b**) Optimized and improved motor mounts. (**c**) Topologically optimized wing ribs. (**d**) Optimized and improved wing ribs.

Figure 18a,c give the design results of the topology optimization of the motor seat and reinforcing ribs of the prototype and the optimal material distribution of the motor seat mass and the reinforcing ribs at the smallest time under the given constraints. The optimization yields a clearer shape of the motor seat and reinforcing wing rib parts. In the actual manufacturing process, the feasibility of parts processing should be taken into account, in addition to the assembly process, since the mounting holes need to withstand the transferred loads, so it is necessary to retain a certain amount of material to ensure its strength. Taking these factors into consideration, the optimized structure of the mitigation groove of the motor seat and wing rib is improved again: firstly, the irregular shape of the edge of the mitigation groove needs to be smoothed and processed, so as to make it easy to process and to prevent stress concentration, and then the mitigation groove with less material removal needs to be deleted appropriately according to the demand. The modified parts are shown in Figure 18b,d.

**Figure 19:**
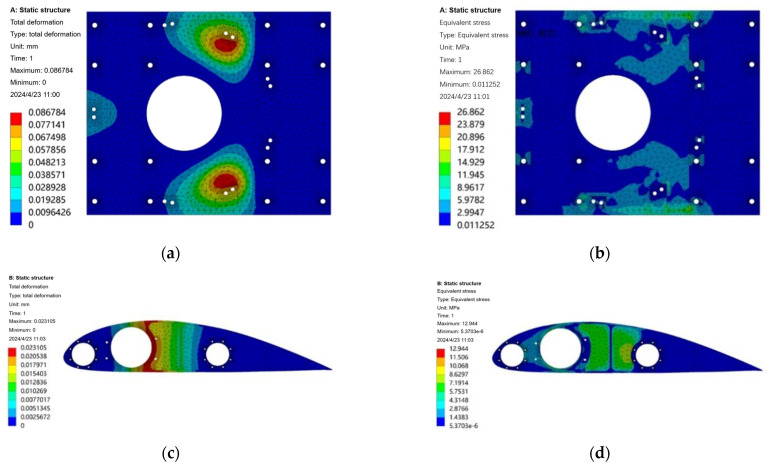
Static strength analysis of optimized member. (**a**) Optimized motor seat deformation cloud. (**b**) Optimized motor seat stress cloud. (**c**) Optimized wing rib deformation cloud. (**d**) Stress cloud of optimized wing ribs.

The improved distribution of the mitigation slots of the motor seat and wing ribs refers to the results of the topology optimization, retaining the material on the main load-bearing part and the optimized mitigation slots of smaller sizes, smoothing the irregularities in the shape of the mitigation slot edges after the material is removed, and retaining the material in the position of the structural mounting holes to a certain extent. The optimized model is subjected to strength verification by using static finite element analysis to analyze the motor mount and wing ribs again under the same load and constraint conditions to verify the strength changes, and the results obtained are shown in Figure 19. The maximum deformation of the motor seat after topology optimization and design modification is obtained to be 0.08 mm, and the maximum stress is 26.86 MPa, which occurs at the bolt hole position of the motor connection. The maximum deformation of the optimized wing rib is 0.02 mm, the maximum stress is 12.94 MPa, which mainly occurs at the contact surface of the main beam and the secondary beam, the maximum equivalent stress value of the optimized motor seat and the wing rib is far less than the fatigue strength limit of the material, and the optimization result meets the experimental requirements. Theoretically, the constraints can be changed to continue the optimization, but considering the need to ensure the structural rigidity and reserve a certain safety factor, the optimization is not continued.

By assigning the material density to the component models before and after optimization in CATIA P3 V5-6R2022 software, it can be calculated that the weight of a single motor seat before optimization is 5.071 kg, and the weight after optimization is 2.234 kg, which is 55.94% lower than that before optimization; the weight of a single reinforced airfoil rib is 3.485 kg before optimization and 1.693 kg after optimization, which is 51.42% lower than that before optimization; and the initial weight of a single normal airfoil rib is 1.706 kg, and the weight after optimization according to the optimization path is 0.845 kg, which is 50.46% lower than that before optimization. The result is shown in Table 4.

According to the analysis of the optimization results, the structural strength and stiffness of the new motor mount and wing ribs are in line with the design requirements, the distribution of the relief grooves is reasonable and easy to process and manufacture, the weight of the parts has been reduced by more than 50%, which is a total of 14.424 kg, and the overall weight of the prototype has been reduced to a large extent.

## 4. Vibration Characterization of the Prototype

### 4.1. Modal Analysis of Prototype

The prototype in the work, the main tail rotor motor, and propeller in the work will produce periodic excitation force, so they are the main source of the prototype body, which is subjected to stable forced vibration, with the motor and the propeller rotating to produce the excitation frequency if the same with the prototype body’s intrinsic frequency. There is a danger of resonance, not conducive to the flight control of the prototype, that will accelerate the fatigue of the machine body, and in serious cases, it may directly damage the airframe, resulting in uncontrollable dangers. Therefore, it is necessary to carry out a dynamic analysis of the airframe to prevent resonance. In this study, the intrinsic frequency of the test aircraft and the external excitation frequency are to be calculated so that the intrinsic frequency of the airframe deviates from the external operating frequency, in which the frequency of the motor and propeller excitation sources involved are calculated as follows:

The formula for calculating the excitation frequency of the motor:

(19) $$f_{a}=n\cdot P/60,$$

$f_{a}$ is the motor excitation frequency; n is the motor speed; and P is the pole pair number of the motor.

There are two main propeller excitation frequencies: propeller shaft frequency, caused by mechanical imbalance, and leaf frequency, caused by an inhomogeneous flow field. The axis frequency calculation formula is as follows:

(20) $$f_{b}=n/60,$$

$f_{b}$ is the propeller shaft frequency; n is the propeller speed. Leaf frequency calculation formula is as follows:

(21) $$f_{c}=N\cdot n/60,$$

$f_{c}$ is the propeller blade frequency; N is the number of propeller blades.

The excitation frequencies of the motors and propellers of the main and tail power systems of the prototype at different rotational speeds can be found by using Equations (19)–(21).

Modal analysis is mainly used to determine the intrinsic frequency, damping ratio, and vibration mode of the structure. According to the analysis results, the vibration characteristics of the structure can be understood, and the response of the structure at a specific frequency can be predicted. In order to avoid resonance when designing the structure, it is necessary to keep the modal frequencies of the structure away from the operating frequency of the mechanical equipment.

In this study, free modal analysis and constrained modal analysis are carried out on the structure of the prototype, and the modal solution is carried out by the chunked Lansos method in Workbench. First of all, in the free modal analysis of the prototype structure, the prototype is suspended in the air, so the free boundary conditions are imposed, so that the modal solution of the prototype body structure is carried out under the unconstrained and unloaded conditions, the modes with the first 80% of the effective participating mass are selected for the analysis, and the modes with intrinsic frequency values and the mode shape characteristics of each order are obtained based on the modal vibration pattern cloud diagrams of the first twelve orders of the modes of the present study (Table 5).

From the results of free modal analysis, it can be seen that the first six orders of the test aircraft body of the calculated intrinsic frequency are very small, and in the first three orders of the intrinsic frequency or even zero, there is no change in the vibration pattern. The average rotational speed of the tail rotor is 3000 RPM, the excitation frequency generated by the motor is 1050 Hz, the propeller shaft frequency is 50 Hz, and the propeller blade frequency is 100 Hz, which are all higher than the first 12 orders of the intrinsic frequency of the airframe. The average speed of the main rotor motor is 1800 RPM, the excitation frequency generated by the motor is 300 Hz, which is much higher than the first 12 orders of the intrinsic frequency of the airframe, the propeller blade frequency is 90 Hz, the propeller shaft frequency is 30 Hz, and the shaft frequency is closer to the 10th and 11th orders of the intrinsic frequency of the airframe. Therefore, it can be initially judged that the vibration characteristics of the airframe in free mode is often the low-valence mode that plays a relatively large influence, the influence of the higher-order modes is smaller, and there will not be any phenomenon of resonance during the flight of the test aircraft because the excitation frequency generated by the rotation of the motor and the propeller are overlapped with the structure’s intrinsic frequency, but it will also be determined later through the harmonic response analysis to further determine whether there will be a resonance danger.

In this study, a constrained modal analysis is also carried out on the structure of the test aircraft. The test aircraft body structure designed in this paper is not only used for the flight test of the UAV but also to fix the whole body on the ground for the ground performance test, to verify the remote control communication function of the control system, and to test the dynamic response of the power system. Before performing the modal analysis, considering that the prototype needs to be fixed on the ground for the ground performance test, it is necessary to impose the actual constraints, and the boundary conditions are applied to the model of the prototype body in Workbench to solve the vibration response characteristics of the machine body in the actual work. The steps of material assignment and meshing are the same as in the free modal analysis of the airframe, but the difference is that the landing gear of the airframe is fixed in the place of contact with the ground. Similarly, the modes with 80% of the effective participating mass of the airframe structure are selected for analysis, the first twelve modes of the airframe are still selected for analysis under the constraints, and the intrinsic frequency values of the modes as well as the vibration characteristics of the modes of each order are obtained in Table 6 according to the vibration cloud diagrams.

From the constrained modal analysis results, it can be seen that the first three orders of the intrinsic frequency are no longer zero, and there is a change in the vibration pattern. The fourth order to the sixth order of the modal vibration pattern has also changed; the first six orders of the modal vibration pattern changes and landing gear constraints related to the state of the latter orders of the vibration pattern are the same with the intrinsic frequency increases, the vibration pattern superposition of the relative complexity. During the ground performance test, the tail rotor is not involved in the test, so the excitation frequency generated by the tail rotor motor and propeller is not taken into account; the rotational speed of the main rotor motor needs to be gradually increased from 200 RPM to 2200 RPM, and each time the speed is increased by 100 RPM, the minimum excitation frequency generated by the motor is 33.33 Hz, which is close to the 8th-order intrinsic frequency of the airframe. And the excitation frequency generated by the motor speed is 50 Hz after the motor speed reaches 300 RPM, which has already been increased by 50 Hz. After the motor speed reaches 300 RPM, the excitation frequency is 50 Hz, which is already higher than the first twelve orders of the intrinsic frequency of the airframe; the excitation frequency generated by the propeller is close to the intrinsic frequencies of the first twelve orders of the constrained modes of the airframe. Therefore, it can be initially concluded that the intrinsic frequency of the airframe and the excitation frequency generated by the propeller are close to each other, and there is a possibility of resonance, but the resonance of the structural structure is not only related to the intrinsic frequency but also to the mode shapes; therefore, the subsequent harmonic response analysis is also used to determine whether there is a risk of resonance of the airframe under the constrained state.

### 4.2. Tester Body Harmonic Response Analysis

**Figure 20:**
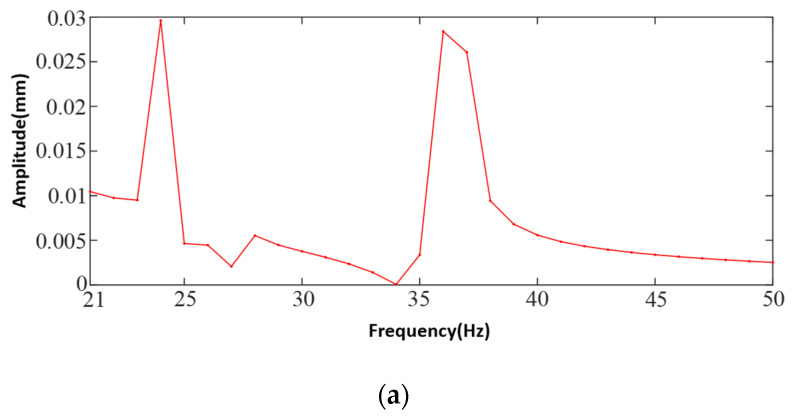

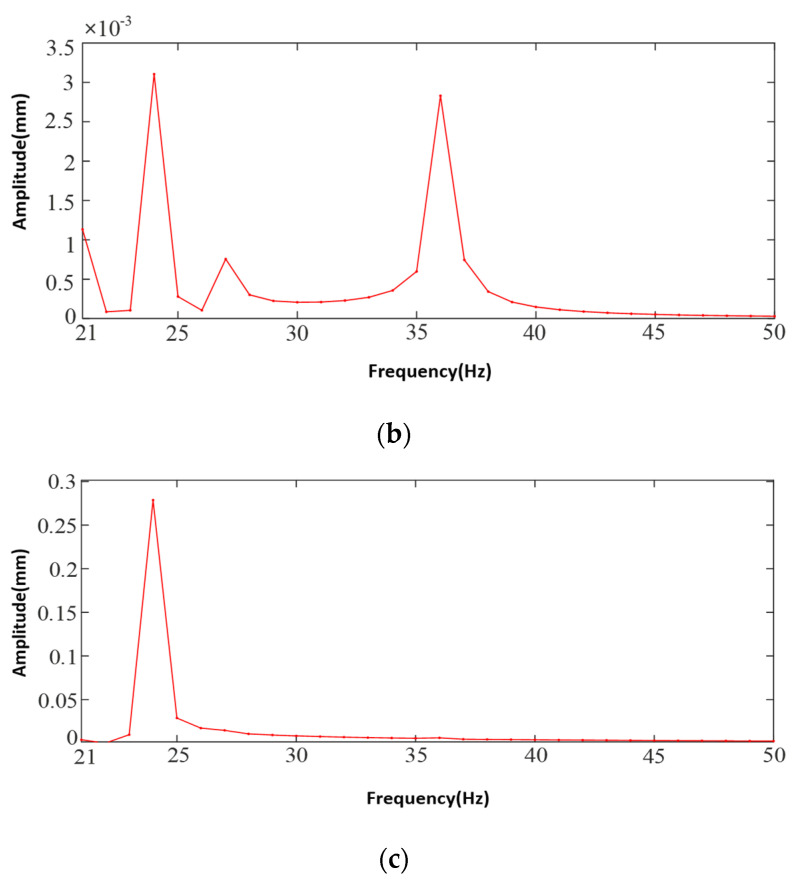
Harmonic response analysis in free mode. (**a**) Amplitude–frequency curve in x-direction. (**b**) Amplitude–frequency curve in y-direction. (**c**) Amplitude–frequency curve in z-direction.

**Figure 21:**
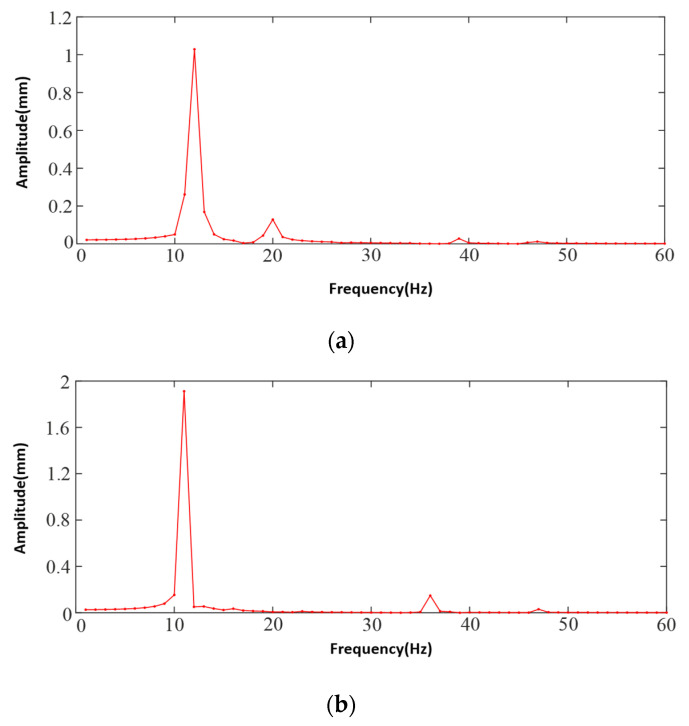

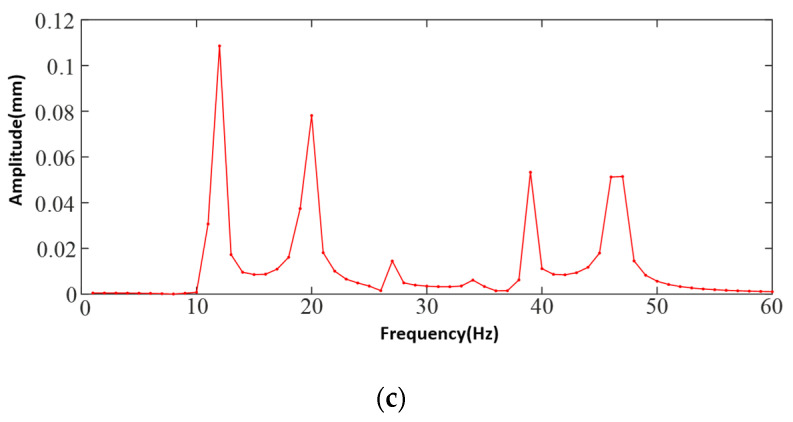
Harmonic response analysis under constrained modes. (**a**) Amplitude–frequency curve in x-direction. (**b**) Amplitude–frequency curve in y-direction. (**c**) Amplitude–frequency curve in z-direction.

Harmonic response analysis calculates the response curves of deformation, stress, and strain of the structure in different frequency ranges, which can obtain the vibration danger point of the mechanical structure, and the frequency range needs to be determined according to the intrinsic frequency and excitation frequency of the mechanical structure. The modal analysis before the harmonic response analysis provides important information about vibration characteristics for the harmonic response analysis. This study analyzes the dynamic response of the body structure under the action of sinusoidal harmonic excitation load, analyzes and solves the “amplitude-frequency” response curve of the body, and analyzes the vibration response characteristics of the body according to the deformation of the body at the frequency corresponding to the maximum displacement in the response curve. This study combines the modal superposition method in Workbench to analyze the body of the prototype, according to the free modal analysis of the body’s intrinsic frequency, and the propeller excitation frequency is set to (20~50) Hz, to set the harmonic response frequency range and to determine the frequency of the solution. The excitation vibration is a torsional vibration; the vertical, transverse, and radial sinusoidal harmonic excitation load is applied to the motor seat and due to harmonic analysis, is a linear proportional relationship, so the load is applied to the motor seat. Since the harmonic response analysis is linear proportionality, the load amplitude size is 16 N for the main rotor and 5 N for the tail rotor; according to the intrinsic frequency of the airframe and the excitation frequency of the propeller obtained from the constrained modal analysis, the harmonic frequency range is set, the solution frequency is set to (0~60) Hz, and the sinusoidal harmonic loads are applied at the motor base in the pendant, transverse, and radial directions at the same time. Also, the load amplitude size is taken to be 16 N. After the above work is completed, the harmonic analysis is performed. Harmonic response analysis is carried out to obtain the results of the harmonic response of the body in free and constrained modes. In free mode, the amplitude–frequency curves in x, y, and z directions are shown in Figure 20a–c under sinusoidal load excitation in vertical, lateral, and radial directions. The amplitude–frequency curves of the body in x, y, and z directions under sinusoidal load excitation in the vertical, lateral, and radial directions in the constrained mode are shown in Figure 21a–c.

Analyzing the harmonic response characteristics in the free mode from Figure 20, the excitation frequency points with large value changes in the x-direction are 24 Hz, 36 Hz, and 37 Hz. While the curve gradually tends to flatten out after 37 Hz, the frequency at the maximum amplitude is 24 Hz, and the maximum amplitude is 0.029 mm; the excitation frequency points with large value changes in the y-direction are 21 Hz, 24 Hz, 27 Hz, and 36 Hz. After 36 Hz gradually declined, where the frequency at the maximum amplitude is 24 Hz, the largest value is 0.0031 mm; z-direction change in the larger value of the excitation frequency point is 24 Hz, the largest value of 0.27 mm, and after 24 Hz the curve gradually tends to be smooth. When the excitation frequency is 24 Hz, the three directions of x, y, and z vibration have the greatest impact, and the z-axis direction of the vibration amplitude of the maximum value is 0.28 mm. Though the three directions of the amplitude–frequency curve can be seen in the 50 Hz before the existence of vibration danger, the amplitude of the smaller value will not have an impact on the prototype; the 50 Hz after the curve amplitude is gradually converging to zero. Therefore, it can be assumed that there is no danger of resonance of the machine body in the free mode.

The harmonic response characteristics in the constrained mode are analyzed in Figure 21. In the harmonic response analysis based on the free mode of the body, the excitation frequency points with large value changes in the x-direction are 12 Hz, 20 Hz, 39 Hz, and 47 Hz, and there are fluctuations between (34~48) Hz, with the frequency at the maximum amplitude of the curve at 12 Hz, and the maximum amplitude is 1.03 mm; the y-direction shows large value changes. The excitation frequency points with larger value changes in the y-direction are 11 Hz, 36 Hz, 40 Hz, and 47 Hz, also fluctuating between (34~48) Hz, where the frequency at the maximum value of the magnitude of the curve is 11 Hz, with the largest value of 1.91 mm; the excitation frequency points with larger value changes in the z-direction are 12 Hz, 20 Hz, 39 Hz, and 47 Hz, and the frequency at the maximum value of the curve magnitude is 12 Hz, with the largest magnitude reaching 0.11 mm.

In summary, from the three directions on the amplitude–frequency curve, it can be seen in 11 Hz and 12 Hz, respectively, the x direction and y direction will produce a larger amplitude, while the other frequency points of the amplitude of the smaller, three curves are at 50 Hz after the gradual convergence to zero. The experimental machine works as long as to ensure that its speed does not stay in the dangerous frequency for a long time to avoid the danger brought about by resonance, which requires that the experimental machine does not appear in the dangerous frequency near the speed frequency of the stable work, which can be controlled by the input command of the control terminal, so there is a need to adjust the speed of the command to avoid the emergence of the 11 Hz and 12 Hz excitation frequency or reduce the excitation frequency to avoid the frequency of the time to avoid the danger caused by the resonance. There is danger due to resonance.

## 5. Prototype System Integration and Control Scheme Optimization

**Figure 22:**
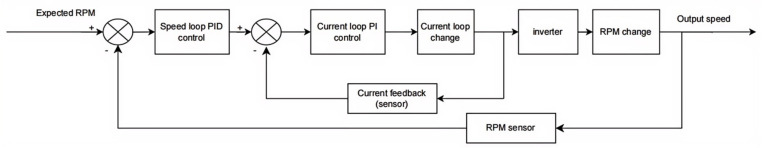
Structural diagram of current loop, speed switched series PID control.

**Figure 23:**
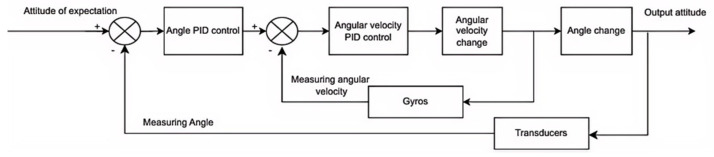
Angle, angular velocity series PID control structure diagram.

The research in this chapter mainly focuses on the selection and control of the system, because the prototype of this research belongs to a large UAV, different from the small UAVs that are more common nowadays, and the prototype of this research is to be applied to the practice of engineering projects, so it is a pioneering idea to apply the dual-series PID control to the tilt-rotor vertical takeoff and landing UAV. One is the tandem PID control with the current loop as the inner loop and the velocity loop as the outer loop, as shown in Figure 22, and the other is the position-velocity tandem PID control with the attitude-transformation angular velocity as the inner loop and the angle as the outer loop, as shown in Figure 23. For the large tilt-rotor vertical takeoff and landing UAV in this study, the dual series PID control method can make the system respond quickly and also meet the strong anti-jamming ability. In contrast, the traditional PID control method is weak in anti-interference ability, the application algorithm of adaptive PID control method in large tilt-rotor vertical take-off and landing UAV is still to be perfected, the system of large tilt-rotor vertical take-off and landing UAV is complex, and the corresponding adaptive PID control method is not as fast as the dual-serial PID control method.

The test system of the prototype is mainly composed of a simulation verification module and a physical flight verification system. The simulation verification module carries out attitude control simulation, and the physical flight test obtains flight data to provide guidance for control design. The simulation verification module consists of a command module, controller, pneumatic module, and six-degree-of-freedom model. The command module sends out commands, the PID controller processes and calculates the control commands, the pneumatic grinding block calculates the force and torque, and ultimately the six-degree-of-freedom model calculates the Eulerian angle of the prototype attitude information. The physical flight test system consists of a ground station, remote control, ground equipment box, and the prototype. The ground station monitors the state of the prototype, the remote control can send control commands to the prototype, and the ground equipment box is equipped with digital receiving and transmitting radios. To realize the ground station and the remote control and for the flight control system on the prototype to transmit the information, the ground station needs to obtain the position and speed information processed by the gyroscope and speed sensor feedback after the controller. The ground station receives the position and speed information from the gyroscope and speed sensor after being processed by the controller.

The prototype itself consists of a power system, control system, and body structure. This section mainly reviews the complete design of the power system and control system of the prototype.

### 5.1. Power System Design

The power system should satisfy the relevant tests such as pull force and torque. The whole weight of the vertical take-off and landing UAV is 350 kg, and each main propeller power system needs to provide at least 175 kg of pulling force. At the same time, it is required that the test aircraft hovering time is at least 7 min, so it is necessary to select a reasonable propulsion system and function. The key components of the power system include four parts: propeller, motor, electronic governor, and battery.

**Figure 24:**
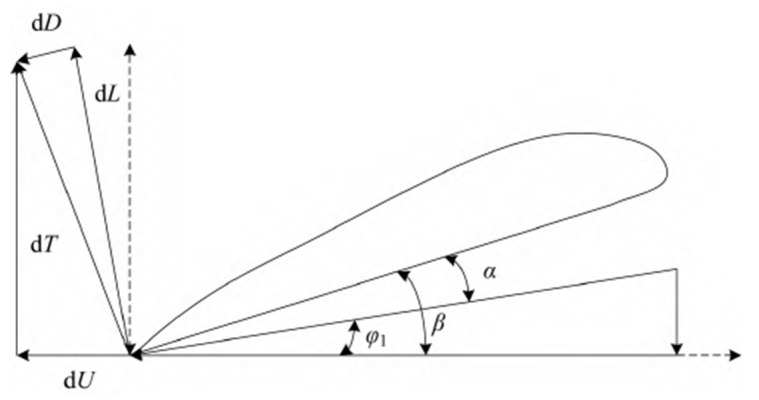
Forces and velocities exerted on propeller blade elements.

There are two kinds of propellers: variable pitch and fixed pitch. To analyze the dynamic model of the variable pitch propeller, the experimental conditions of the test site where this prototype is located have small wind speeds. It can be approximated that the propeller’s operating state is in a static thrust state, based on the theory of leaf vein modeling of the variable pitch propeller. As Figure 24 shows, the propeller is at the radius of the corresponding leaf vein force state and velocity state. It indicates the pitch angle corresponding to the variable pitch propeller of this study, the angle of approach corresponding to the variable pitch propeller, and the drag force and lift force on a single vane.

Obtained from the basic foliation theory,

(22) $$W_{0}=\sqrt{v^{2}+{\left(rw\right)}^{2}},$$

v is the incoming flow velocity; w is the pitch paddle rotational speed; and $W_{0}$ is the combined velocity of the paddles by the airflow

(23) $$dD=\frac{1}{2}C_{D}\rho W_{0}^{2}c(r)dr,$$

(24) $$dL=\frac{1}{2}C_{L}\rho W_{0}^{2}c(r)dr,$$

ρ is the air density; $C_{L}=C_{L\alpha }(\alpha -\alpha_{0})$ is the lift coefficient; $C_{D}=C_{D0}+kC_{L\alpha }^{2}{\left(\alpha -\alpha_{0}\right)}^{2}$ is the drag coefficient; $\alpha_{0}$ is the zero lift head angle; and c(r) is the blade chord length at the paddle, which can usually be expressed as $c(r)=k_{1}r$.

From the decomposition of the forces, the circular dF and dT tensile forces on the leaf vein are

(25) $$dF=dL\sin\varphi_{1}+dD\cos\varphi_{1},$$

(26) $$dT=dL\cos\varphi_{1}+dD\sin\varphi_{1}$$

Combining Equations (23)–(25), the torque $dM_{p}$ applied to the variable pitch at r can be obtained:

(27) $$dM_{p}=\frac{1}{2}\rho W_{0}^{2}k_{1}r^{2}[C_{L}\sin\varphi_{1}+C_{D}\cos\varphi_{1}]dr,$$

The torque $M_{p}$ applied to the whole propeller is obtained by integrating Equation (27):

(28) $$\begin{array}{l} M_{p}=N_{b}\cdot \int_{0}^{R}dM_{p}=N_{b}-\frac{1}{2}\rho k_{1}\cdot \\ \{C_{La}(\alpha -\alpha_{0})\sin\varphi_{1}+[C_{D0}+kC_{La}^{2}{\left(\alpha -\alpha_{0}\right)}^{2}]\cos\varphi_{1}\}\cdot \\ \int_{r}^{R}\left[{\left(rw\right)}^{2}+v^{2}\right]\times r^{2}dr \end{array},$$

$N_{b}$ is the number of paddle blades; r is the radius of the propeller disk.

For the tensile force T applied to the whole propeller, it is obtained by integrating Equation (26):

(29) $$\begin{array}{l} T=N_{b}\cdot \int_{0}^{R}dT=N_{b}\cdot \frac{1}{2}\rho k_{1}\cdot \\ \left\{C_{L\alpha }(\alpha -\alpha_{0})\cos\varphi_{1}+[C_{D0}-{kC}_{L\alpha }^{2}{\left(\alpha -\alpha_{0}\right)}^{2}]\sin\varphi_{1}\right\}\cdot \\ \int_{0}^{R}[{\left(rw\right)}^{2}+v^{2}]\cdot rdr \end{array},$$

Assuming that the basic parameters of the propel k, $k_{1}$, $C_{L\alpha }$, and the external environmental parameters ρ are constants that do not vary with time, the accuracy of the model is verified by the ground test bed, and the incoming flow velocity v=0, which is known for the current modeling, is $\alpha_{1}=0,\beta =\alpha ,\alpha_{0}=0$.

Equations (28) and (29) can be deformed as follows:

(30) $$M_{p}=\frac{1}{10}\rho N_{b}k_{1}C_{D0}R^{5}w^{2}+\frac{1}{10}\rho N_{b}k_{1}C_{L\alpha }^{2}R^{3}\beta^{2}w^{2}=q_{1}w^{2}+q_{2}\beta^{2}w^{2},$$

(31) $$T=\frac{1}{10}\rho N_{b}k_{1}C_{L\alpha }R^{4}\beta w^{2}=q_{3}\beta w^{2},$$

**Figure 25:**
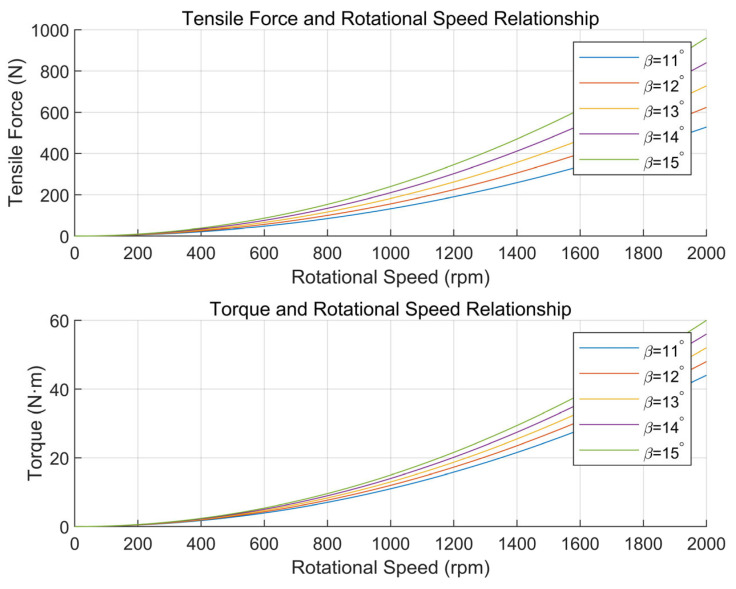
Effect of rotational speed and pitch angle on pulling torque.

From the formula, it can be concluded that the torque and tensile force of the propeller are only related to the pitch angle and rotational speed. According to this theoretically derived formula, in order to fit the relationship between propeller tension and torque and rotational speed and pitch angle in a real scenario, assuming that the propeller rotational speed ranges from 0 to 2000 RPM, and for the unknown parameter to be identified, the example value $q_{1}=1\times 10^{-6},q_{2}=1\times 10^{-6},q_{3}=1\times 10^{-6}$ is given, which should be replaced by the real value when the actual scenario is in progress, and the tension and torque on the propeller plotted in conjunction with this modeling is shown in Figure 25. It can be seen that tension and torque are proportional to the square of rotational speed and increase with the increase in rotational speed, and they increase with the increase in pitch angle.

So the variable pitch propeller can flexibly adjust the pulling force and torque, which can meet the more variable UAV load situation and complex environment. This design adopts a three-blade propeller with a diameter of 1.9 m, which is a variable pitch propeller, and the maximum pulling force that this propeller can provide meets the design index.

Brushless DC motor has high efficiency, low loss, and at the same time, it is better than other motors in terms of reliability, stability, adaptability, and life span, and the diameter of the propeller belongs to the large-size propeller, so the selection of brushless motor is a large torque and low kv value motor, so the motor of the tail power system adopts the EA160 motor, the motor of the main power system adopts EMRAX-268 motor (EMRAX doo, Kamnik, Slovenia), and the external dimension of the main power system motor is 268 mm. Also, the peak torque is 500 N-m to meet the required torque parameters of this propeller selection.

The selected main and tail powertrain motors are both brushless DC motors, both of which require electronic speed controllers to work with them. The main function of the electronic speed controller is to convert the DC input into a three-phase input for the motor, and at the same time, to regulate the speed of the motor. According to the selected EMRAX-268 motor and EA160 motor parameters, the maximum current of the motor is 320 A. Therefore, we choose the ESC DTI-HV500 with a maximum continuous current of 400 A as the main power system ESC and the EP-330A-HV ESC for the tail power system.

Battery selection should take into account factors such as lightweight, high efficiency, and environmental protection, and the use of lithium batteries as the energy source of the UAV is a suitable choice. For battery selection red dot electric 12S-8000mAh-25C, of which 12S indicates that each battery is connected in series by 12 lithium batteries, the nominal voltage of a single lithium battery is 3.7 V, the fully charged state is 4.2 V. 8000 mAh, which refers to the capacity of the battery, and the discharge can last 1 h at 8000 mA. 25C indicates the discharge multiplier, which means that the maximum discharge current of the battery is 25 times the capacity, and it can discharge at 200 A for 0.04 h. A total of 15 pieces of this type of battery are connected in series for the single-side main power system, and 2 pieces of this type of battery are connected in series for the single-side tail power system.

### 5.2. Control System Design

#### 5.2.1. Control Object Modeling

**Figure 26:**
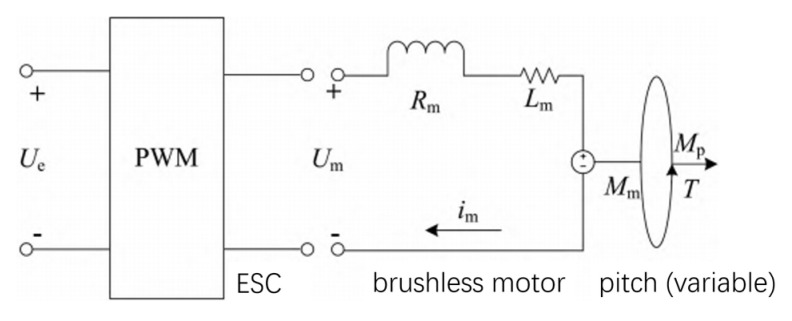
ESC-motor equivalent circuit model.

The power system of this study uses a brushless DC motor as the drive motor and is equipped with a corresponding electronic governor to control the motor. Figure 26 shows the equivalent circuit corresponding to the outer rotor brushless DC motor and its electronic governor. The input voltage of the motor $U_{m}$ is regulated by controlling the battery voltage $U_{e}$ input to the electronic governor through PWM wave signals, and the duty cycle of the control signal σ ranges from 0 to 100%, corresponding to the minimum and maximum power of the motor. The relationship between the battery voltage $U_{e}$, duty cycle σ, and input voltage $U_{m}$ of the motor is as follows:

(32) $$U_{m}=\sigma U_{e},$$

$R_{m}$ and $L_{m}$ are the resistance and inductance of the motor. The stator generates a counter-electromotive force $U_{\alpha }$ when the motor is in operation, and the current in the stator armature interacts with the permanent magnets to produce an electromagnetic torque $M_{m}$. This is obtained from the potential balance equation and the torque balance equation:

(33) $$U_{m}=i_{m}R_{m}+L_{m}\frac{di_{m}}{dt}+U_{\alpha },$$

(34) $$J\dot{\omega }=M_{m}-f\omega_{0}-M_{p},$$

J represents the total rotational inertia of the motor rotor and pitch paddle; f represents the friction coefficient of the motor; the reverse electromotive force is proportional to the rotational speed $U_{\alpha }=k_{n}\omega_{0}$; and the torque is proportional to the current $M_{m}=k_{T}i_{m0}$.

The combined Equations (32)–(34) give the expression of the dynamic model of the ESC and the motor whose input consists of the rotational speed $w_{0}$, the PWM signal σ, and the variable pitch propeller as the sole load of the motor:

(35) $$A_{1}\frac{d^{2}\omega_{0}}{\;dt}+B_{1}\frac{d\omega_{0}}{dt}+C_{1}\omega_{0}+D_{1}M_{p}=\sigma ,$$

The parameters to be recognized in Equation (35) are as follows:

(36) $$\left\{\begin{array}{l} A_{1}=\left(L_{n}J\right)/\left(k_{T}U_{e}\right)\\ B_{1}=\left(L_{n}f\right)+(J/\left.k_{T}U_{e}\right)\\ C_{l}=\left(f+k_{m}k_{T}\right)/\left(k_{T}U_{e}\right)\\ D_{l}=\left(R_{m}+L_{m}\right)/\left(k_{T}U_{e}\right) \end{array}\right.,$$

The motor and propeller used in the electric propulsion system under study are directly connected by hardware and have the same rotational speeds $w=w_{0}$. Substituting Equation (30) into Equation (35) yields a nonlinear electric propulsion model whose input consists of pitch angle β, PWM signal σ, and output consists of motor speed $w_{0}$. Considering that the inductance of the brushless DC motor is very small and has little effect on the accuracy of this modeling, assumption $L_{m}\approx 0$, the simplified model is obtained for this modeling:

(37) $$A_{2}\dot{\omega }+B_{2}\omega +C_{2}\omega^{2}+D_{2}\omega^{2}\beta^{2}=\sigma ,$$

The parameters to be recognized are, respectively, as follows:

(38) $$\left\{\begin{array}{l} A_{2}=J/\left(k_{T}U_{e}\right)\\ B_{2}=\left(f+k_{T}k_{m}\right)/\left(k_{T}U_{e}\right)\\ C_{2}=\left(R_{m}q_{1}\right)/\left(k_{T}U_{e}\right)\\ D_{2}=\left(R_{m}q_{2}\right)/\left(k_{T}U_{e}\right) \end{array}\right.,$$

Since the battery voltage of the lithium battery before the cut-off voltage is relatively small in the whole electric propulsion system operation, $A_{2},B_{2},C_{2},D_{2}$ in Equation (37) can be used as the constants to be recognized, and $q_{1},q_{2},q_{3}$ are the relevant parameters about the paddle. So the nonlinear dynamic model equation of the electric drive pitch is as follows:

(39) $$\left\{\begin{array}{l} A_{2}\dot{\omega }+B_{2}\omega +C_{2}\omega^{2}+D_{2}\omega^{2}\beta^{2}=\sigma \\ M=q_{1}\omega^{2}+q_{2}\beta^{2}\omega^{2}\\ T=q_{3}\beta \omega^{2} \end{array}\right.,$$

Equation (39) is a nonlinear system model with a first-order mixing term, and special points are usually chosen to simplify it for this complex model structure. When the torque Mp≈0 is measured in the experimental data at the pitch angle β=0, the rotational speed equation of Equation (37) can be simplified to a first-order inertial link with the duty cycle σ as the input and the rotational speed w as the output, since the nonlinearity of Equation (37) is generated by the torque of the propeller:

(40) $$A_{2}\dot{w}+B_{2}w=\sigma ,$$

The gain K and time constant T of the system can be obtained by applying a step signal to the input of the variable pitch electric propulsion system by means of time-domain identification, acquiring the rotational speed data of the system, and importing the input signal and the output rotational speed data of the system into the MATLAB R2023a system identification toolbox. Equation (41) is the transfer function obtained by stepping the duty cycle from 10% to 20%:

(41) $$G=\frac{257.8}{0.311s+1},$$

We can know the parameters from Equation (41): $A_{2}=1.2\times 10^{-3},B_{2}=3.9\times 10^{-3}$.

Since the model identification of the controlled object electric propulsion system results in a section of inertial links, PID control is used to regulate the dynamic response of the system. Because the traditional PID control method is weak in anti-interference ability, and because the application algorithm of the adaptive PID control method in the field of unmanned aerial vehicles is very complicated, this research adopts the serial PID control method, which is strong in anti-interference ability and simple in realization.

#### 5.2.2. Control System Design and Parameter Tuning

The control system adopts the DSP28377D control board, which contains a number of commonly used modules such as a communication module, motor drive module, relay module, digital tube display module, and signal processing module, which can realize the control system required by the design. This design only serves as an experiment for vertical takeoff and landing, and the main control and regulation parts involved are prototype attitude control regulation and current loop and speed loop control regulation. Based on the electric propulsion system identification model, the dual-cascade PID control method is adopted. The cascade PID control of the current and velocity loops uses the current loop as the inner loop and the velocity loop as the outer loop to realize the stable control of the UAV motor speed. The prototype adopts serial PID to realize attitude control with attitude change angular velocity control as the inner loop and angle control as the outer loop. The control signal input form adopts two signal input forms: one is to use the ground station to input the rotational speed command and receive the signal with the receiver to realize the control of the motor speed; the other is to use the remote control to input the rotational speed signal. The receiver also receives the rotational speed control command to realize the control of the motor speed.

In this study, for the regulation of PID parameters, the experimental method of control variables is used. Compared to the way of determining the PID parameters through simulation and analysis, the control variable method does not rely on the mathematical model of the system and does not require an accurate mathematical model, while being able to have a direct adaptation to the real system and avoiding the poor performance of the parameters in the real system is due to the idealized assumptions in the simulation. As a rule of thumb, the current loop is first regulated to ensure that the system can achieve the required stabilized power. Then the speed ring is regulated to optimize the speed response. After the current and speed loops are regulated, the actual propeller speed of the prototype and the attitude of the prototype are then tracked and tested experimentally according to the serial PID control. This approach is well thought out and reasonable with a relatively small number of experiments.

**Figure 27:**
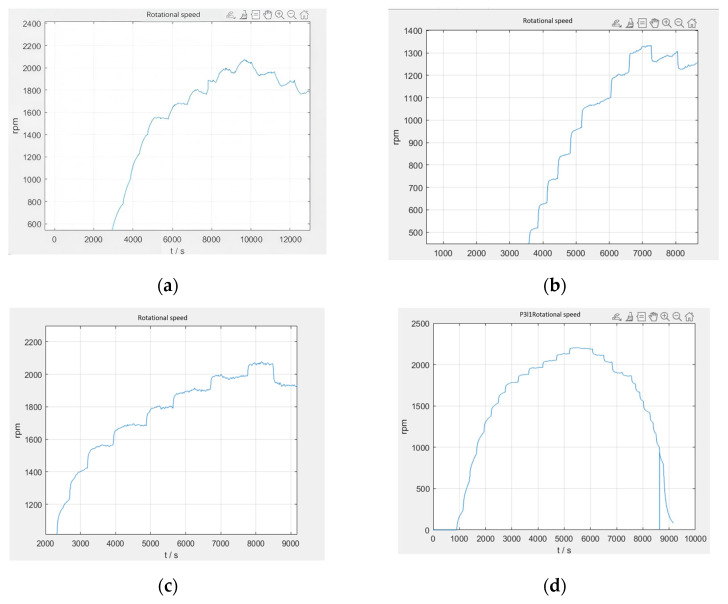
Comparison of speed profiles with different controller parameters (simulation results). (**a**) P = 0.4, I = 0.6. (**b**) P = 2, I = 0.01. (**c**) P = 3, I = 0.5. (**d**) P = 3, I = 1.

The current loop parameter control first adopts PID control, and by optimizing the parameters of the PID controller, the current loop conforms to the current release situation to ensure that the system achieves the required stable power. As the inner loop, the main function of the current loop is to control the motor current according to the regulation signal of the speed loop. The current loop measures the motor current and compares it to the regulation signal to generate a PWM duty cycle output signal. The PWM duty cycle signal is finally fed to the inverter, which carries out PWM modulation of the motor power supply and finally realizes the speed control of the motor. The current loop is controlled by PI, firstly, the controller parameters are not modified, the default parameters are used for simulation experiments, and then the controller parameters are optimized according to the experimental effect, so as to obtain the simulation response curve of the system under different parameters, as shown in Figure 27.

Figure 27a shows the response curves of the default parameters of the system for the first experiment, which are relatively less valuable to analyze in the low-speed region due to the faster speed of command sending and the consideration of battery power saving. Therefore, the main focus is on the high-speed region. In the high-RPM segment, the speed response is significantly slower after each step speed command is given, especially in the region close to 1600 RPM, where the speed shows a climb up to the target speed. This clearly indicates that the current loop proportionality coefficient has insufficient step capability and mainly relies on the integration effect of the integration link. In order to optimize the system performance, the proportional parameter should be increased while the integral parameter should be decreased.

Based on the analyzed results in Figure 27a, further experimental observations were carried out after increasing the proportional parameter in the current loop parameters to 2 and reducing the integral link to a minimum value of 0.01. As shown in Figure 27b, the effect of increasing the proportional parameter is positive in terms of speed feedback. The speed feedback curve rises almost vertically at each step, which indicates that the response is accelerating and the system performance is improved, but there is still a lack of step capability. Therefore, while continuing to increase the proportionality coefficient, it is considered that the integral parameter can be increased a little bit appropriately.

According to the results of the Figure 27b experimental plotting analysis, the current loop proportional parameter is set to 3, and the integral parameter is set to 0.5, as shown in Figure 27c. The observation of the speed feedback curve found that the speed response has been relatively ideal, each response slightly overshooting the situation, which led to decisions to focus on the next time for the integral parameter to make changes.

According to the analysis results of Figure 27c, it can be seen that this current loop parameter has already made the speed curve response speed and stability reach a better balance, but in order to further optimize the current loop parameters, the proportional parameter of the current loop is kept unchanged, and the integral parameter is increased. It is found that the enhancement of the integral link in the process of increasing the integral parameter helps to control the change in the current in a better way, and at the same time the speed curve also becomes smoother. This shows that the stability of the system response is improved. Therefore, the current loop parameters are adjusted to a proportional coefficient 3 and integral coefficient 1, as shown in Figure 27d, and this parameter combination shows a better performance in the experiment, which not only ensures the fast response of the rotational speed but also realizes the stable control of the current.

**Figure 28:**
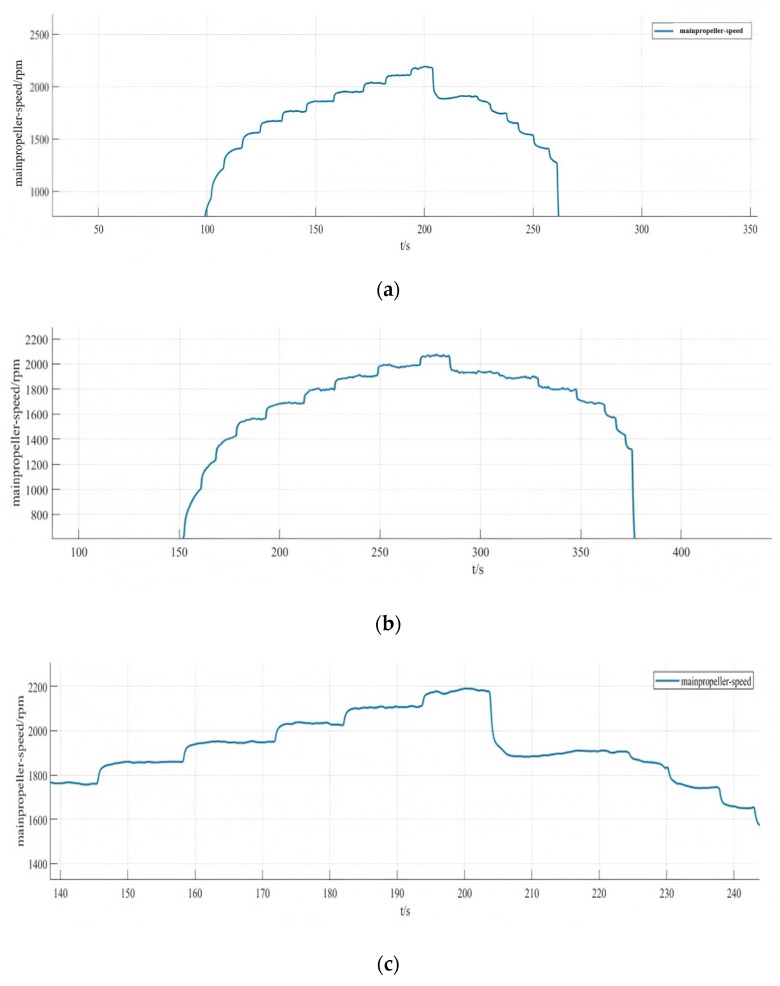
Velocity feedback curve of velocity loop with different controller parameters (simulation results). (**a**) P = 0.4 i = 0 d = 0.002. (**b**) P = 0.8 i = 0.02 d = 0.002. (**c**) P = 0.8 i = 0.02 d = 0.004.

After adjusting the current loop parameters, the system can have a stable output power. Then optimize the design of the control parameters of the speed loop. This regulation uses PID regulation. There are three regulation links: proportional, integral, and differential. Set the initial controller parameters for the proportional parameter at 0.4, integral 0, and differential 0.002 for a set of experiments, based on this simulation analysis to obtain the response data and further optimization of the parameters and to obtain several adjustments of the response curve in Figure 28.

Figure 28a shows that the speed curve has improved responsiveness during the response but still does not reach the target value, and the steady state error is large when the 2100 RPM command is given. The response speed is only 2050 RPM, which is not in accordance with the expectations. To solve this problem, the proportionality parameter is increased, and a little integral link is added. The increase in the proportional parameter helps to increase the response speed of the system, and the integral link helps to reduce the steady-state error.

Under the analysis results of Figure 28a, the speed loop proportional parameter is set to proportional parameter 0.8, integral 0.02, differential 0.002, and the speed feedback curve is shown in Figure 28b, when increasing the proportional parameter after the responsiveness to the improvement, and at the same time, the steady state error decreases, and the speed curve fluctuates in the range a little bit. The next time the adjustment will be increased by the differential parameter appropriately to carry out the optimization.

On the basis of the analytical results of Figure 28b, after increasing the parameter of the differential link to 0.004 alone, the rotational speed feedback curve is shown in Figure 28c, which is still relatively stable even after amplification, with a fluctuation range of 5 RPM, and the rotational speed loop adjustment up to this point has already met the demand of the subsequent experiments.

**Figure 29:**
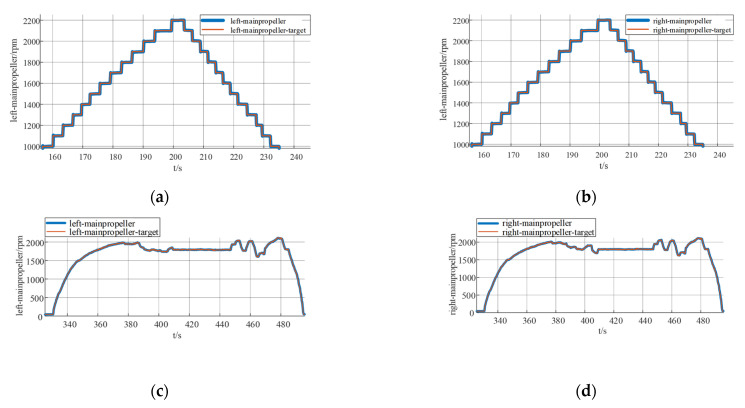
Dynamic response co-conditioning test results for real experiments (actual experimental results). (**a**) Ground station commands left main slurry speed. (**b**) Ground station commands right main slurry speed. (**c**) Remote control command left main pulp speed. (**d**) Remote control command right main pulp speed.

After the controller optimization is completed, the actual control performance of the prototype is tested. Because there are a lot of interference signals in the prototype control system, the traditional PID controller has limited anti-interference ability. In order to improve the anti-interference ability of the prototype control system, and in order to avoid the problem of slow response speed brought by the overly complex algorithm to the large unmanned aerial vehicle system, this study adopts the serial PID control method to control the system. The remote control terminal inputs control commands to the UAV, mainly inputting command speed commands, and the telemetry terminal is used to receive feedback data, which mainly includes real-time values such as motor speed, voltage, current, temperature, etc. The remote control terminal is Futaba, which is used to control the UAV. The remote control is a Futaba T18SZ model (Futaba Corporation, Mobara, Japan) with 2.4 GHz frequency, up to 18 channels and a matching receiver. The results are shown in Figure 29. From curves Figure 29a,b, it can be seen that, when the ground station sends out the speed command, the response performance of the motor meets the requirements, the speed curve tracks well, and the speed fluctuation range is within 5 RPM, so the speed of the prototype is stabilized at 1800 RPM, and the maximum speed can be up to 2200 RPM. It can be seen from Figure 29c,d that the rotational speed curve of the propeller is highly consistent with the required rotational speed curve of the propeller, which can accurately control the rotational speed of the motor through the remote control commands and realize the stable flight of the prototype.

**Figure 30:**
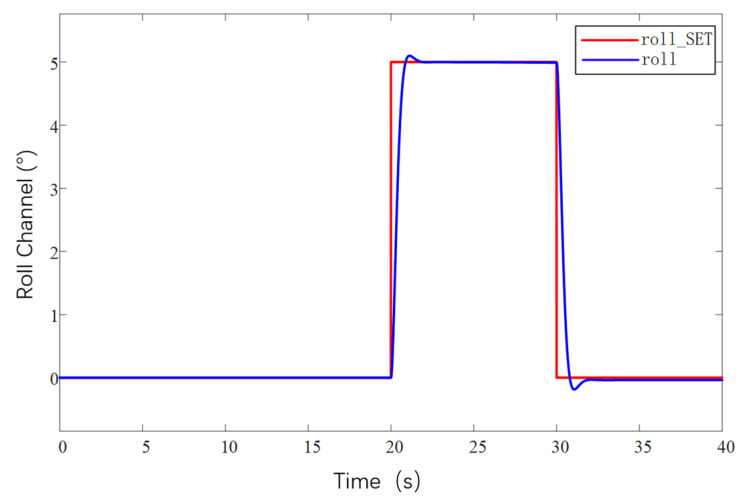
Roll angle control response curve (simulation result).

**Figure 31:**
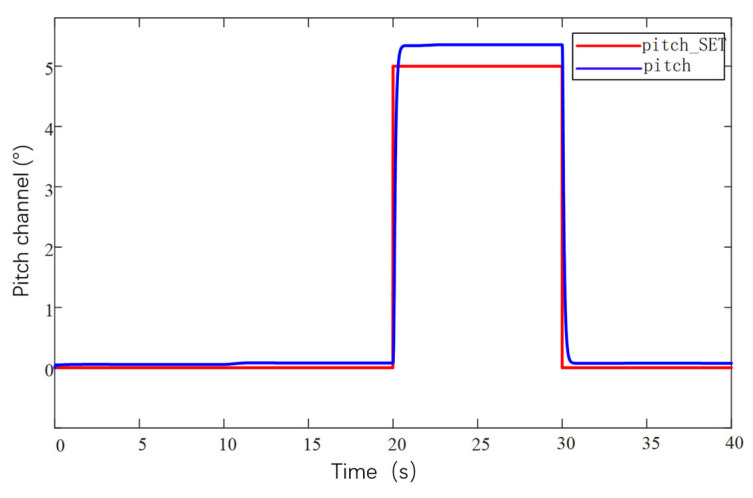
Pitch angle control response curve (simulation result).

**Figure 32:**
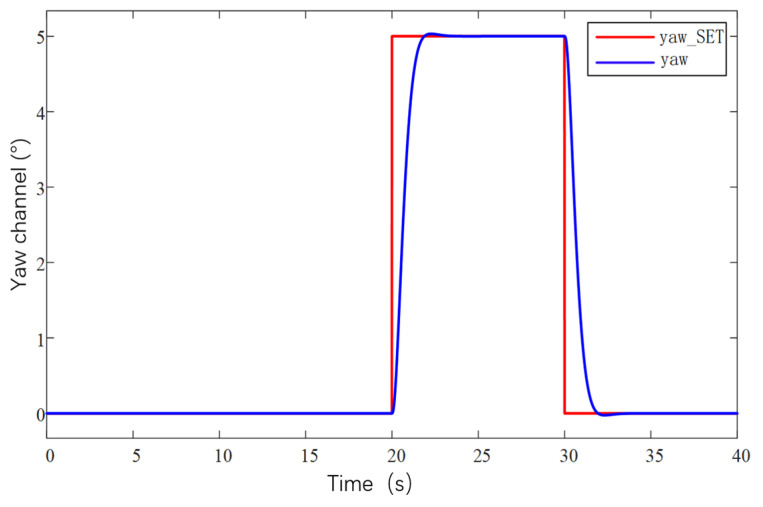
Yaw angle control response curve (simulation result).

After the propeller speed tracking experiment was completed, the attitude simulation control of the test airplane was carried out in combination with the second serial PID control method. The serial PID for attitude control takes the angular velocity of attitude change as the inner loop and the angle of attitude change as the outer loop. The simulated tracking of the pitch, roll, and yaw angles of the prototype is shown in Figure 30, Figure 31 and Figure 32. They are the roll angle step response curve, pitch angle step response curve, and yaw angle step response curve, respectively.

From Figure 30, it can be seen that the response time of the roll angle response curve is delayed by 0.8 s, and there is some overshooting, with an overshooting amount of 0.09, all within the acceptable range; from Figure 31, it can be seen that the pitch angle response time is fast, reaching the desired value in only 0.3 s, but there exists a certain amount of steady-state error, with a steady-state error value of 0.3, which is within the error range; from Figure 32, it can be seen that the response curve of the yaw angle time delay is greater than the roll angle. The delay time is 1.5 s, within the acceptance range of yaw angle 5°, and there are no overshoot and steady state error. All three response curves have certain delays and errors, but they are within the acceptable range, so the simulation results meet the design requirements. Meanwhile, the simulation results also verify the excellent performance of the dual-serial PID control in this study from the theoretical level, and the dual-serial PID control method is theoretically very suitable for large tilt-rotor vertical takeoff and landing UAVs.

**Figure 33:**
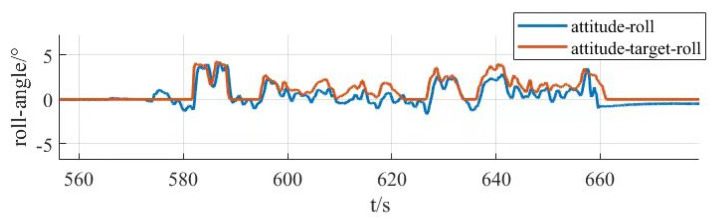
Roll tracking curve (actual experimental results).

**Figure 34:**
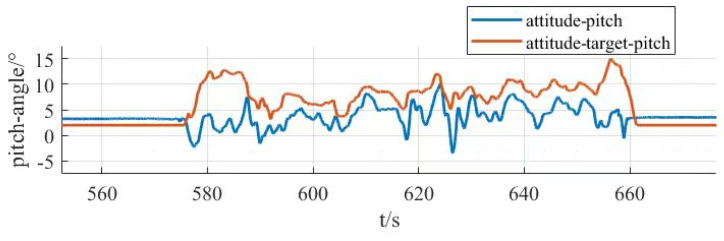
Pitch tracking curve (actual experimental results).

**Figure 35:**
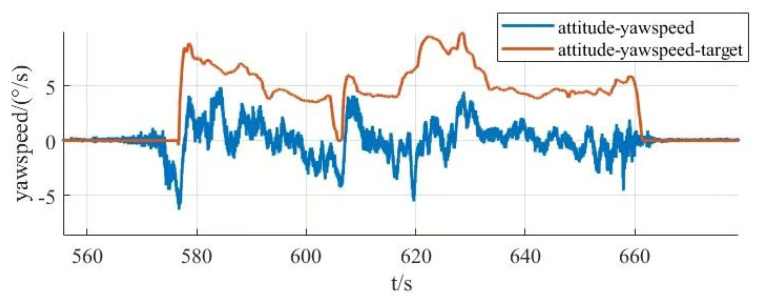
Yaw tracking curve (actual experimental results).

After the prototype attitude tracking simulation is completed, it is tested for actual low-altitude flight attitude tracking. The desired attitude output was achieved by the action between the propeller motors. The flight control data was downloaded after the completion of the experiment, and the tracking profiles shown in Figure 33, Figure 34 and Figure 35 were obtained, showing the tracking profiles of the test airplane during roll, pitch, and yaw hover experiments.

The roll control of the prototype is carried out through the differential of the two main rotors, keeping the speed of the left main rotor unchanged and increasing the speed of the right main rotor, so that the tension generated by the right main rotor increases, the prototype realizes the left roll, and on the contrary realizes the right roll, the tail rotor, is not involved in the roll control, and its speed remains unchanged during the process. The pitch control of the prototype is carried out through the difference between the main and tail rotors, keeping the speed of the two main rotors unchanged, increasing or decreasing the rotational speed of the tail rotor makes the lift generated by the tail rotor change, and the prototype realizes the change in pitch attitude; the yaw control of the prototype is carried out through the difference between the two tail rotors, keeping the speed of the left tail rotor unchanged and increasing the rotational speed of the right tail rotor to make the pull generated by the right tail rotor increase, the prototype realizes that the right yaw, and conversely the left yaw, and the main rotor are not involved in yaw control, and the speed remains unchanged in the process. There is a small jitter before takeoff, and it gradually stabilizes after takeoff.

The tester roll control is good, the roll angle curve and the roll angle target curve trend are consistent, the maximum error is within 10%, within the acceptable range, the pitch angle and yaw angle curve and the target curve trend are consistent, the response speed is good, but there is a large error. Analyzing the reason for the large error, the test aircraft has been in low-altitude horizontal flight, the propeller and the ground will form a complex flow field, and there is the formation of the ground effect. The ground effect causes an increase in the lift generated by the rotor, and the smaller the relative height of the rotor to the ground, the more pronounced the ground effect is, the relative height being the ratio of the height above the ground to the diameter of the rotor. The test aircraft pitch is controlled by the rotational speed change in the tail rotor, the main rotor is subject to the ground effect is more pronounced, so the tail rotor to increase the lift is not enough to meet the requirements of the change in the pitch attitude; the test aircraft yaw is accomplished through the tail rotor’s differential, and the actual change in yaw curve value is much smaller than the target curve value. The yaw is accomplished by the differential movement of the tail rotor, and the actual change in yaw in the curve diagram is much smaller than the target curve value, which may be due to the fact that the rotational speed of the tail rotor is not enough to increase the lift, and on the other hand, the angle between the axis of the tail rotor and the z-axis of the fuselage coordinate system does not satisfy the requirements. Also, the y-axis direction of the y-axis is not enough.

In this section, the power system and control system of the prototype were designed. The propeller motor speed control and the flight attitude control of the prototype were carried out, and the relevant control data were obtained through the ground station and the remote control, respectively, which provided the experimental data to guide the research of the tilt-rotor concept UAV.

## 6. Conclusions

Based on the reality that the research of tilt-rotor vertical take-off and landing UAVs mostly stays in the theoretical research and verification, this paper designs a new tilt-rotor vertical take-off and landing UAV prototype and obtains the relevant design data of tilt-rotor vertical take-off and landing UAV through experiments, which provides a reliable data support and data reference for the future research of this type of UAV. This paper establishes a dynamic model of tilt-rotor vertical take-off and landing UAV according to the flight principle of the quadrotor UAV and completes the overall design of the prototype, material selection, and dimension design according to the structural form and design index of the UAV; analyzes the load and deformation response of the prototype according to the static analysis of finite element, and the maximum deformation of the prototype occurs at the edge of the wing beam and the main motor seat after the force is applied, with the maximum deformation value of 57.1 mm, to achieve less than 3% of the design requirements. The maximum equivalent force of the prototype body structure is concentrated in the scaffolding fasteners, the maximum equivalent force value of 379.21 MP, less than its minimum strength limit of 450 MPa, the maximum stress concentrated in the 6061 and 7075 aluminum alloy tubes of 142.2 MPa, less than its minimum ultimate strength of 240 MPa and 455 MPa. The landing gear of the prototype is optimized by the unified objective method, and the beam length and angle of the landing gear after optimization are 579.15 mm and 123.9°, respectively. Combining with the topology optimization module in the Workbench, the motor seat and wing ribs are optimized by using the poi density method and the static structure module, the maximum deformation of the motor seat after topology optimization and modification of the design is 0.08 mm, the maximum stress is 26.86 MPa, the maximum deformation of the motor seat is 0.02 mm, and the maximum deformation of the wing rib is 0.02 MPa. The maximum deformation of the wing ribs is 0.02mm, and the maximum stress is 12.94 MPa, which meets the requirements of this study. The vibration response characteristics are analyzed under the free mode, the vibration response characteristics are analyzed under the constrained mode, and combined with the harmonic response analysis, it is concluded that the rotational speed command should avoid the occurrence of the excitation frequency of 11 Hz and 12 Hz or reduce the excitation frequency to stay time to ensure the vibration safety performance of the prototype, according to the prototype flight. According to the flight load of the test aircraft, the power system of the test aircraft is constructed, and the control system is optimized. Through the actual flight test, the propeller speed tracking fluctuation of the test aircraft is within 5 RPM, and the error of the roll attitude of the test aircraft is within 10%. However, the test site of the experiments in this paper is limited, and for the sake of the experimental safety, the test aircraft only carries out the experiments in low altitude, the experimental results are affected by the ground effect, and the pitch attitude and yaw attitude control errors are larger; therefore, the future will be further developed. Adaptive PID control will be further introduced in the future. Our research will refine the adaptive PID algorithm on the basis of ensuring the response rate of a large tilt-rotor vertical takeoff and landing UAV and conduct flight experiments at higher altitudes to eliminate the influence of ground effects. On this basis, future research will shift the test site to low-altitude and high-altitude areas to enhance the prototype’s ability to adapt to the environment and achieve more precise control.

## Figures and Tables

**Table 1:** Design indicators.

Parameters	Numerical Value	Parameters	Numerical Value
fuselage length	3000 mm	Tail rotor wheelbase	2190 mm
fuselage width	548 mm	Main tail rotor X-direction wheelbase	1763.7 mm
fuselage height	645 mm	Maximum weight of the drone	350 kg
wingspan	4045 mm	Maximum main rotor power	160 kW
tail spread	2260 mm	Tail rotor power	18 kW
main rotor wheelbase	3695.4 mm	Thrust ratio	1.3

**Table 2:** Deformation and stress of landing gear under different working conditions.

Working Condition	Maximum Deformation (mm)	Maximum Equivalent Force (Mpa)
working condition 1	3.34	186.65
working condition 2	3.45	264.19

**Table 3:** Comparison before and after optimization.

Optimization Variables	Bow-Beam Pole Length L (mm)	Bow-Beam Angle α (°)	Mass m (kg)	Maximum Equivalent Force (Physics) σ (Mpa)
pre-optimization	600	120	5.795	616.54
post-optimization	579.15	123.9	5.758	412.46

**Table 4:** Quality comparison before and after optimization.

Name	Quality Before Optimization (kg)	Quality After Optimization (kg)	Optimization Percentage
Electric motor base	5.071	2.234	55.94%
Reinforced wing ribs	3.485	1.693	51.42%
Ordinary wing ribs	1.706	0.845	50.46%

**Table 5:** Free modal analysis results of the prototype body.

Order (Number of Step)	Intrinsic Frequency (Hz)	Vibration Pattern
1	0	/
2	0	/
3	0	/
4	6.70	Rotational deformation of the fuselage around the z-axis of the fuselage coordinates
5	8.52	Rotational deformation of the fuselage around the x-axis of the fuselage coordinates
6	9.40	Rotational deformation of the fuselage around the y-axis of the fuselage coordinates
7	14.68	Torsional deformation of the tail section of the fuselage around the x-axis
8	21.13	Wing and tail twist in opposite directions around the z-axis, tail ends twist up and down irregularly
9	24.07	Fuselage twisted from side to side, wing ends swinging up and down in deformation
10	27.13	Fuselage twisting from side to side, tail swinging up and down in deformation
11	36.25	Fuselage twisted and deformed fore and aft, tail wing swung up and down.
12	36.81	Fuselage twisted and deformed left and right, tail wing swung up and down and deformed

**Table 6:** UAV body constraint modal analysis results.

Order (Number of Step)	Intrinsic Frequency (Hz)	Vibration Pattern
1	10.93	The fuselage rotates and deforms around the landing gear.
2	12.09	The fuselage rotates around the landing gear and deforms forward and backward.
3	12.67	Wing and tail swing up and down.
4	15.79	Wing twisting forward and backward, tail swinging up and down, and deformation.
5	19.69	The fuselage moves forward and backward, and the tail swings up and down.
6	22.71	The fuselage is twisted and deformed from side to side, and the tail is swinging up and down irregularly.
7	26.82	The fuselage swings up and down at the wing and up and down in the opposite direction at the tail.
8	34.52	Fuselage and tail swing up and down.
9	36.07	Fuselage twisted from side to side, wings and tail swinging up and down, and deformed.
10	38.84	Fuselage twisted forward and backward, wing reverse-twisted forward and backward, tail swinging up and down.
11	46.48	Fuselage twisted up and down, wings twisted back and forth, up and down.
12	46.92	Combined left-right and up-down twisting and deformation of the fuselage.

## Data Availability

Data are contained within this article.
